# Network pharmacology-based approach to investigate the molecular targets of essential oil obtained from lavender for treating breast cancer

**DOI:** 10.1016/j.heliyon.2023.e21759

**Published:** 2023-11-08

**Authors:** Guzhalinuer Maitisha, Junhao Zhou, Yan Zhao, Shuxia Han, Youyun Zhao, Ablikim Abliz, Guangzhong Liu

**Affiliations:** a Clinical Laboratory, Hubei Provincial Hospital of Traditional Chinese Medicine, Hubei Province Academy of Traditional Chinese Medicine, Wuhan 430061, China; b School of Laboratory Medicine, Hubei University of Chinese Medicine, Wuhan 430065, China; c Department of Gastrointestinal Surgery, Hubei Provincial Hospital of Traditional Chinese Medicine, Hubei Province Academy of Traditional Chinese Medicine, Wuhan 430061, China

**Keywords:** Lavender essential oil, Breast cancer, Network pharmacology, Molecular targets, PI3K-AKT signaling pathway

## Abstract

Lavender essential oil (LEO) is known for its medicinal use in the development of pharmaceuticals. Further investigations were demonstrated that LEO has many biological properties including apoptosis. However, The anti-breast cancer activity and mechanism of LEO are still unclear. we aim to elucidate the elusive anti-breast cancer activity and mechanism of LEO by unveiling the intricate molecular targets that it engages with, thereby priming it for effective therapeutic intervention against breast carcinoma. In this paper, we extracted LEO from lavender and analyzed it's chemical constituents by using hydro-distillation and gas chromatography-mass spectrometry (GS-MS/MS) method, respectively. The active components against breast cancer and it's molecular targets were selected and biological process, molecular function, cellular component and involving pathways were evaluated via network pharmacology approach. Cell viability, apoptosis and cell cycle assay were used to evaluate anti-breast cancer effect of LEO. Employing the western blotting method to validate target protein expression following LEO treatment *in vitro*. We found the 21 effective components and 213 drug-disease common targets of LEO. Amoung them, 7 active components and 19 targets were identified as potential therapeutic targets. Gene ontology results revealed that the drug-disease common targets of LEO were mainly distributed in membrane region, involved in peptide-tyrosine phosphorylation, and primarily associated with protein tyrosine kinase. We also found that drug-disease common targets might contribute to the regulation of PI3K-AKT signaling pathway by using KEGG pathway analysis. Besides, our study demonstrated reduced cell viability, induced apoptosis in MCF-7 and MDA-MB-231 treated with LEO while cell cycle arrest was not altered. The AKT1 expression down-regulated while PIK3CA expression was increased in both cell lines. Our findings indicate that LEO has the ability to induce apoptosis by modulating the expression of PI3K-AKT signaling pathway in these cell lines.

## Graphical abstract

**Figure:**
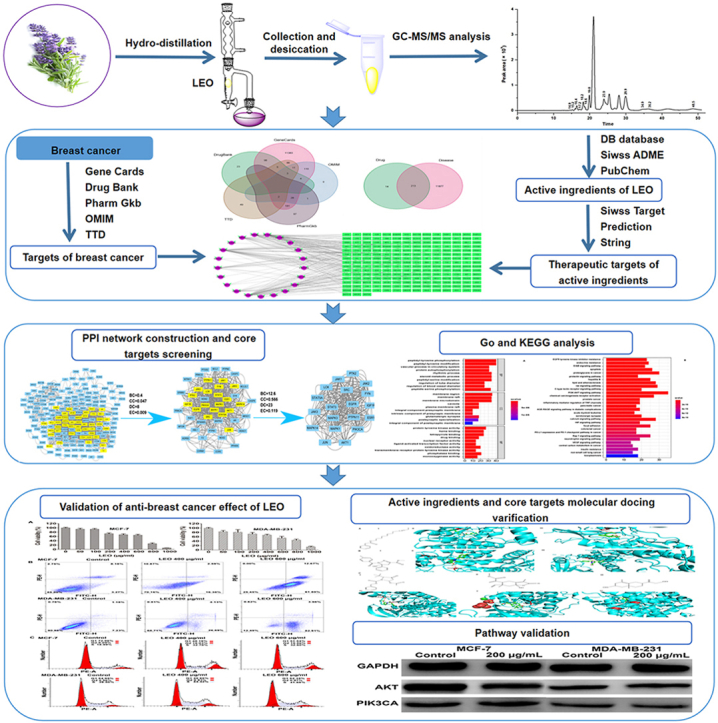

## 1 Introduction

Breast cancer in female is the major malignancy in the world due to its high morbidity and cancer related deaths [1]. Breast cancer were frequently diagnosed and tending to even younger ages in recent years. Breast cancer treated with conventional methods such as radiotherapy, chemotherapy and hormone therapy has limited therapeutic effect and reduce life quality of patient due to their adverse reactions [2]. In addition, several risk factors including multi-drug resistance, high costs, and ineffective responses mitigate therapeutic effectiveness and increase the mortality of patients [3]. Therefore, the search for new anticancer substances is essential for breast cancer treatment.

Essential oils (EOs) extracted from aromatic herbs and medicinal plants used as promising drug for prevention and treatment of various human diseases in several traditional medicine due to their several qualities such as antitumor, anti-inflammatory, antibacterial and antioxidant [4,5]. Their cytotoxic potentials can overcome some of challenges in the cancer treatment. Lavender (*Lavandula angustifolia Mill., Lamiaceae*) is mainly originated from Mediterranean, Middle East and now is cultivated worldwide [6]. Lavender essential oil (LEO), a natural sterile hybrid obtained by lavender has been used as important herbal medicine in many regions since the ancient times [7]. It has been widely used in many fields including aromatherapy, cosmetics, perfumes, massage oil, detergents, food processing, shampoo and tea in addition to as drugs [8]. Further investigations on LEO biological activities were showed anti-oxidant, anxiolytic and anti-depressive, anti-inflammatory, analgesic, anti-fungal and bactericidal, apoptosis and autophagy properties [9,10]. Researchers have make an effort on it's anti-cancer effect and demonstrated that LEO exhibits much more effect on various cancer cells and induce apoptosis such as lung adenocarcinoma, cervical carcinoma, breast cancer, glioma and prostate cancer [11]. Although many research studies have reported the biological activity of LEO, there are very few detailed publications about the action of LEO on breast cancer cells.

Recently, network pharmacology has testified as a promising method for clarifying the correlation between targets, diseases and drugs, meanwhile analyzing results from a holistic perspective [12]. Network pharmacology has been used to visualize drug-disease-target networks of compounds used in traditional chinese medicine (TCM) [13]. Similarly, it may act as a optimal method for selecting the therapeutic target and underlining mechanisms of LEO for breast cancer treatment.

This study aims to evaluate the potential biological targets and signaling pathways of LEO for the treatment of breast cancer through network pharmacological analysis, and then verify its anti-breast cancer effects and therapeutic targets *in vitro*. Our scientific discoveries offer novel opportunities for developing anticancer medications and establish a theoretical foundation for treating breast cancer.

## 2 Materials and methods

### 2.1 Plant preparation

Lavender grown under conventional systems and plastic covering, harvested during the flowering season in Yili, Xinjiang Uyghur Autonomous Region, China, dried in shade, and kept under laboratory conditions at room temperature (25 °C).

### 2.2 LEO extraction

LEO extracted from dried flowers of lavender. Sixty gram samples were powdered and extracted by hydro-distillation using a fat extractor apparatus for 1.5 h at 100 ± 5 °C. The extracted substances were dehydrated by using anhydrous MgSO_4_, pure extracted oils were stored at 4 °C for further experiment.

### 2.3 Determination of the chemical composition of LEO

Filtered dry LEO samples were injected into a gas chromatography-mass spectrometry (GC-MS) system (Agilent 7890A-5975C, USA) with a HP-5MS capillary column (30.00 mm × 0.25 mm × 0.25 μm) for the purpose of volatile compound analysis and identification. The carrier gas used was helium with a flow rate of 1 ml/min, and the split ratio was 1:100. The analysis procedure consisted of follows: (a) maintaining a temperature of 50 °C for 0 min, (b) the temperature rises from 50 °C to 280 °C at a rate of 5 °C per minute and remains constant for a duration of 10 min. For the mass spectrometry detector, an electron impact was used as ionization source with the temperature of 180 °C and the ionization voltage of 70 EV. The injector and detector were operated in 250 °C. The isolates from the gas spectrometry were full scanned (*m*/*z* range of 50∼500) by mass spectrometry. The chromatographic peak area more than 0.12 % was selected and chemical components corresponding to each peak further analyzed by using NIST508 online library.

### 2.4 Screening of targets of chemical components from LEO

Four databases, including PubChem, Swiss ADME, Swiss Target Prediction and String were used to identify the targets of chemical components from LEO. The chemical composition of LEO was identified using the compounds structure and CID number, based on the canonical SMILES ID acquired from the PubChem database.Then, pharmacokinetic parameters namely gastrointestinal absorption (GI absorption), blood brain barrier (BBB), P-glycoprotein substrate (P-gp substrate) and drug likeness (DL) were obtained from Swiss ADME according to smiles ID. The active ingredients of LEO are screened by taking GI absorption is “yes”, BBB is “yes”, P-gp substrate is “no”, drug likeness is “yes” as screening conditions. The corresponding drug targets of the screened chemical components were firstly filtered in Swiss Target Prediction database, and then underwent additional screening in the String database, adhering to the screen condition of “score ≥0.8”.

### 2.5 Screening targets for breast cancer

Five databases including GeneCards, PharmGkb database, Therapeutic Target Database (TTD), OMIM and Drug Bank were serves as an invaluable tool for identifying promising drug targets for breast cancer. All databases expected targets affiliated with breast cancer containing the keyword “breast cancer”. Finally, the target subset predicted from these five databases was consolidated and identified as the cancer target.

### 2.6 Intersection of targets for breast cancer and LEO

The R “Venn” software package (version 4.0.2) was utilized to perform a Venn analysis on the common targets of breast cancer and LEO. The overlapping targets for both bioactive compound and breast cancer were identified as potential targets for LEO in its effort to combat breast cancer. Establish and visualize drug target networks using bioactive compounds and their predictive targets with the help of Cytoscape software (version 3.8.0).

### 2.7 Protein-protein interaction (PPI) network analysis

The PPI of the predicted target can be examined using the String database. An acceptable level of interaction is denoted by applying a cut-off criterion, which requires a minimum interaction score of ≥0.9. The PPI data is then employed in the Cytoscape software (version 3.8.0) to create PPI networks, while maintaining the default settings for all other parameters. PPI networks were topologically calculated and visualized using the CytoNCA plug-in, which included degree centrality (DC), betweenness centrality (BC), eigenvector centrality (EC) and closeness centrality (CC).

### 2.8 Enrichment analysis of gene ontology (GO) and kyoto encyclopedia of genes and genomes (KEGG) pathways

We utilized three software packages, namely ClusterProfiler, Dose and Enrichplot, within the R software (version 4.0.2), to perform enrichment analysis on GO and KEGG pathway. Significance was assigned to GO terms and KEGG pathways with a p-value of ≤0.05. For our further analysis, we selected the foremost 10 GO terms for cellular components (CC), molecular function (MF), and biological process (BP), as well as the top 30 KEGG pathways.

### 2.9 Molecular docking

The 2D structure of the docking compound was sourced from the PubChem Data Bank, and subsequently imported into the Chem BioOffice software (version: 14.0.0.117) to reduce the atomic distance and obtain the ligand file in mol2 format for each compound. The UniProt database provides the accession number of the potential target protein (Homo sapiens). To obtain the 3D structures, the Protein Data Bank database was utilized, and modifications were made using PyMOL software (version: 2.4.0) to eliminate superfluous ligands and water prior to the docking experiment. Autodock Vina software (version: 1.5.6) was used for molecular docking.The binding affinity between the active compound and the target protein was computed, and the optimal poses for these ligands to engage with the amino acid residues having the lowest molecular docking energy were identified.

### 2.10 Cell lines and culture

The Cell Bank of the Type Culture Collection (CBTCC), located at the Chinese Academy of Sciences (Shanghai, China), offers breast cancer cell lines (MCF-7 and MDA-MB-231) that were carefully cultivated in a specific medium known as Dulbecco Modified Eagle Medium (DMEM) (New York, USA). The medium was composed of 10 % inactivated fetal bovine serum (FBS) that had been thoroughly heated, along with penicillin (100 U/mL) and streptomycin (100 U/mL). The cells were placed in an incubator (WCI-180, Wiggens, Germany) at a temperature of 37 °C and maintained in an atmosphere with 5 % CO_2_.

### 2.11 Cell viability assay

The impact of LEO on cell viability was assessed using the MTT assay.The MCF-7 and MDA-MB-231 breast cancer cells were meticulously seeded onto the 96-well plate with an exacting cell density of 5 × 10^3^ cells per well. After a 24-h incubation period, the cells were ready for further analysis. The cells were subjected to treatment with various concentrations of LEO, starting from 50, 100, 200, 400, 600, 800 and 1000 μg/mL for an additional 24-h period. The cells designated as the control were grown in a culture medium containing 0.5 % of dimethyl sulfoxide (DMSO), whereas the cells that were not treated with LEO and DMSO were categorized as the black group. After the prescribed cultivation period, a 10 μL solution of 5 mg/mL MTT reagent was added to each well for the MTT analysis. Subsequently, the substance was incubated for an additional 4 h at a temperature of 37 °C. Then, dissolve the formazan crystals that have formed by adding 100 μL of DMSO, and remove the medium. The absorbance can be measured by utilizing a microplate reader (Bio-Tek Epoch microplate spectrophotometer, Vermont, USA) at the wavelength of 570 nm. The percentage of cell viability was determined by calculating it according to Eq. (1).

Eq. 1 $$\text{Cell}\;\text{viability}\left(\%\right)=\frac{OD_{LEO}-OD_{Blank}}{OD_{Control}-OD_{Blank}}\times 100\%$$

### 2.12 Apoptosis assay

The Annexin V Apoptosis Detection Kit (BD Biosciences, San Jose, CA, USA), which includes fluorescein isothiocyanate (FITC), was employed for analyzing apoptosis. Breast cancer cells are incubated in 6-well plates at a rate of 1 × 10^5^ cells per well for 24 h, and then subjected to LEO treatment at different concentrations (0, 400 and 600 μg/mL). Twenty-four hours after treatment, collect the cells, wash them, and resuspend them in 400 μL of 1 × binding buffer. Add 5 μL of annexin V-FITC conjugate with 5 μL of PI solution to the cell suspension, then incubate at ambient temperature in the dark for 15 min. The cell population undergoing apoptosis, viability and necrosis was then evaluated by means of the flow cytometer's Cell Quest software (Zhongsheng Biosino, China).

### 2.13 Cell cycle assay

Breast cancer cell lines were cultivated in specialized 6-well plates. Subsequently, these cell lines were exposed to varying concentrations (0, 400 and 600 μg/mL) of LEO extracts for 24 h periods. Cell collection and rinsing in ice-cold phosphate-buffered saline (PBS) were conducted. Subsequently, the sample was fixed by treating with 70 % ethanol at 4 °C overnight. Flow cytometry was employed to assess the distribution of the cell cycle following washing with PBS containing 500 μL of PI/RNASE staining buffer. After then, the samples were incubated in the dark at room temperature for 15 min. The Cell Quest software (BD Bioscience, USA) was employed for conducting data analysis.

### 2.14 Western boltting

To prepare MCF-7 and MDA-MB-231 cell samples for analysis, they were rinsed in cold (PBS), and subsequently dissolved using a Radio-Immunoprecipitation Assay (RIPA) kit along with control and 200 μg/mL LEO treatment.The poteins are subject to equal separation through electrophoresis on a 10 % SDS-PAGE gel, and are then transferred to a polyvinylidene fluoride membrane (PVDF). Next, a blocking step is conducted using 5 % skim milk (100 mM NaCl, 50 mM Tris, 0.1 % Tween 20, pH 7.5) in TBST, allowing the proteins to be appropriately examined. To do so, incubate the membrane with primary antibodies against AKT1, PIK3CA and GAPDH at dilution ratios of 1:1000, 1:1000 and 1:2000, respectively, overnight.The membrane, which had been preserved at a temperature of 4 °C, was subsequently cleaned with TBST. Following this, the sample was exposed to cultivation alongside the fitting secondary antibody, which had been conjugated with horseradish peroxidase. Enhanced chemiluminescence (ECL) is used to examine signals. All regents and materials were obtained from Servicebio company (Servicebio, Wuhan, China).

## 3 Results

### 3.1 Determination of active components in LEO

**Fig. 1:**
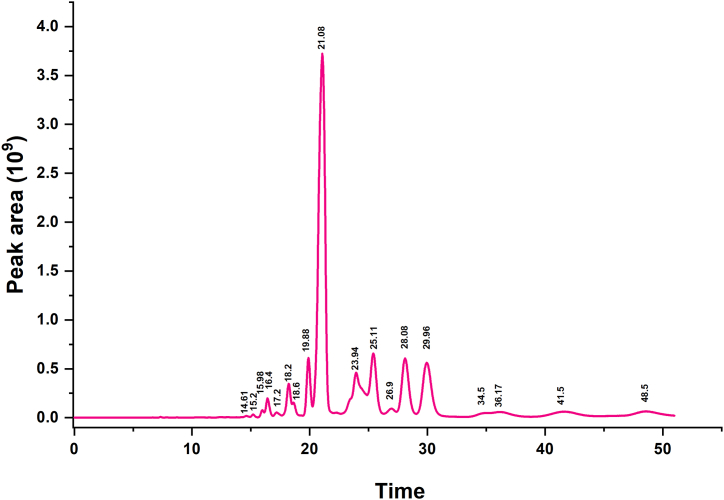
The total ion chromatography of LEO. The LEO components **were determined** in full scan mode **by** using GC-MS/MS.

In this study, we extracted the light yellow LEO and analyzed its effective components by using GC-MS/MS and obtained 12 chromatographic peaks with peak areas greater than 0.12 % (Fig. 1). The ten most likely components corresponding to each chromatographic peak were obtained through DB database search. The first five components with high scores were selected to consideration and among them unsaturated hydrocarbons and organic acids were excluded (Table S1). Then 21 effective components meet the standard of the pharmacokinetic parameters were screened (Table 1).

**Table 1:** The active components of LEO.

Time	CID No	Name	Formula	Canonical SMILES	MW	Drug likeness（DL）	GI absorption（OB）	P-gp substrate	BBB
14.61	562380	1,4-Methano-1H-cyclopenta [*d*]pyridazine, 4,4a,5,7a-tetrahydro-8,8-dimethyl-, (1.alpha.,4.alpha.,4a.alpha.,7a. alpha.)-	C_10_H_14_N_2_	CC1(C2C3CC=CC3C1N=N2)C	162	Yes; 0 violation	High	No	Yes
	576686	2-Pyrazoline-3-carboxylic acid, 5-hydroxy-1-(4-methylbenzoyl)-5-phenyl-, methyl ester	C_19_H_18_N_2_O_4_	COC(=O)C1=NN(C (=O)C2=CC=C(C)C=C2)C(O)(C1)C1=CC=CC=C1	338	Yes; 0 violation	High	No	No
15.2	564243	8-Methylenebicyclo [4.2.0]oct-4-en-3-one	C_9_H_10_O	C=C1CC2C=CC(=O)CC12	134	Yes; 0 violation	High	No	Yes
16.4	560987	3-Thiazolidinecarboxylic acid, 4-(acetyloxy)-2-(1,1-dimethylethyl)-, phenylmethyl ester, 1-oxide, [1R-(1.alpha., 2.beta.,4.beta.)]-	C_17_H_23_NO_5_S	CC(=O)OC1CS(=O)C (N1C(=O)OCC2=CC=CC=C2)C(C)(C)C	353	Yes; 0 violation	High	No	No
	522804	4-Benzyloxyphenylacetonitrile	C_15_H_13_NO	C1=CC=C(C=C1)COC2=CC=C(C=C2)CC#N	223	Yes; 0 violation	High	No	Yes
17.2	557603	3,10-Dioxatricyclo [4.3.1.0 (2,4)]dec-7-ene	C_8_H_10_O_2_	C1C=CC2CC3OC3C1O2	138	Yes; 0 violation	High	No	Yes
	544156	Acrylic acid 5-methylidene-6-heptenyl ester	C_11_H_16_O_2_	C=CC(=C)CCCCOC(=O)C=C	180	Yes; 0 violation	High	No	Yes
18.2	556420	Spiro [cyclopropane-1,6'- [3]oxatricyclo [3.2.1.0 (2,4)]octane]	C_9_H_12_O	C1CC11CC2CC1C1OC21	136	Yes; 0 violation	High	No	Yes
	6673	Dicyclopentadiene diepoxide	C_10_H_12_O_2_	C1C2C3CC4C(C3C1C5C2O5)O4	164	Yes; 0 violation	High	No	Yes
18.6	249955570	7,8-Diazabicyclo [4.2.2]deca-2,4,7,9-tetraen-7-oxide	C_8_H_8_N_2_O	O=N1=NC2C=CC1\C=C/C=C\2	148	Yes; 0 violation	High	Yes	Yes
19.88	556274	Bicyclo [2.2.1]hept-5-en-2-yl-acetaldehyde	C_9_H_12_O	[H]C (=O)CC1CC2CC1C=C2	136	Yes; 0 violation	High	No	Yes
	572048	3-Caren-10-al	C_10_H_14_O	CC1(C2C1CC(=CC2)C=O)C	150	Yes; 0 violation	High	No	Yes
23.94	561932	beta-Terpinyl acetate	C_12_H_20_O_2_	CC(=C)C1CCC(CC1)(C)OC(=O)C	196	Yes; 0 violation	High	No	Yes
29.96	61275	**Nerol oxide**/2H-Pyran, 3,6-dihydro-4-methyl-2-(2-methyl-1-propenyl)-	C_10_H_16_O	CC1=CCOC(C1)C=C(C)C	152	Yes; 0 violation	High	No	Yes
	10819	**Perillyl alcohol**/1-Cyclohexene-1-methanol, 4-(1-methylethenyl)-	C_10_H_16_O	CC(=C)C1CCC(=CC1)CO	152	Yes; 0 violation	High	No	Yes
	561502	Phenylacetic acid, dodec-9-ynyl ester	C_20_H_28_O_2_	CCC#CCCCCCCCCOC(=O)CC1=CC=CC=C1	300	Yes; 1 violation	High	No	Yes
34.95	561486	Tricyclo [4.2.1.0 (2,5)]non-7-en-3-one	C_9_H_10_O	O=C1CC2C1C1CC2C=C1	134	Yes; 0 violation	High	No	Yes
36.17	244005	2-(2-Methylphenyl)propan-2-ol	C_10_H_14_O	CC1=CC=CC=C1C(C)(C)O	150	Yes; 0 violation	High	No	Yes
48.5	555304	alpha.-Phenethyl cyanide, 2-methoxy-6-nitro-	C_10_H_10_N_2_O_3_	CC(C#N)C1=C(C=CC=C1OC)[N+](=O)[O-]	206	Yes; 0 violation	High	No	Yes
	535386	Bicyclo [4.4.0]dec-5-ene, 1,5-dimethyl-3-hydroxy-8-(1-methylene-2-hydroxyethyl-1)-	C_15_H_24_O_2_	CC1=C2CC(CCC2(C)CC(O)C1)C (=C)CO	236	Yes; 0 violation	High	No	Yes
	543063	Sericealactone（5-Benzofuranacetic acid, 2,4,5,6,7,7a-hexahydro-7a-hydroxy-3,6-dimethyl-.alpha.-methylene-2-oxo-6-vinyl-, methyl ester ）	C_16_H_20_O_5_	CC1=C2CC(C (CC2(OC1=O)O)(C)C=C)C (=C)C (=O)OC	292	Yes; 0 violation	High	No	Yes

### 3.2 Using network pharmacological analysis to screen candidate targets for treating breast cancer in LEO candidates

**Fig. 2:**
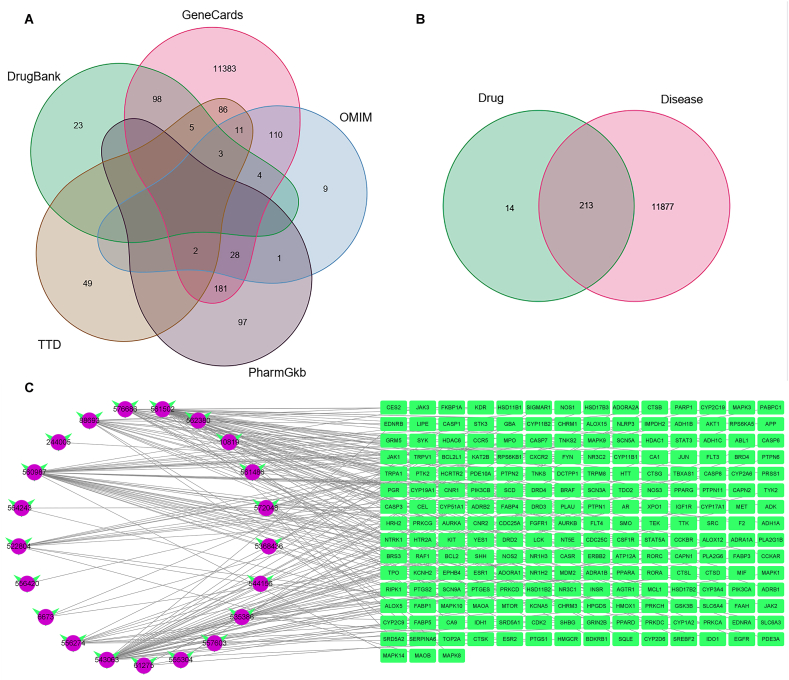
Screening the candidate targets of LEO for treating breast cancer. A: The venn diagram of disease target collected from Drug Bank, OMIM, Gene Cards, TTD and Pharm Gkb; B: The venn diagram of “drug-disease” common targets; C: Network of “LEO active components-drug target”.

The identification of LEO active components potential targets for breast cancer management was made possible by the utilization of PubChem, Swiss Target Prediction and String databases, resulting in the discovery of 228 potential targets (Table S2). Explore potential targets related to breast cancer using databases such as Gene Cards, Pharm Gkb, TTD, Drug Bank and OMIM. The 11911, 309, 158, 216 and 409 potential targets were identified from each of the five databases (Table S3).After eliminating duplicate targets, the ultimate count of potential targets linked to breast cancer was reduced to 12,090 (Fig. 2A). Notably, by utilizing a Venn Diagram, 213 drug-disease common targets were obtained among the 228 potential targets of LEO and the 12,090 potential targets related with breast cancer (Fig. 2B). We used Cytoscape 3.8.0 to construct a potential drug-disease common target network, which was shown in Fig. 2C.

### 3.3 Analysis and screening of core targets in PPI network

**Fig. 3:**
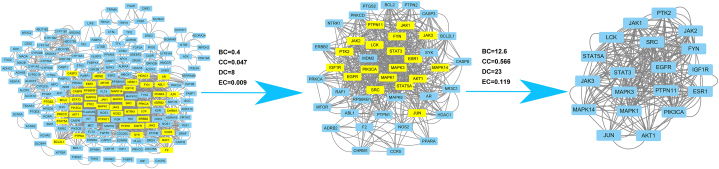
PPI network analysis and screening of 19 core targets.

Next, the interactions and correlation functions among 213 predicted targets were systematically detected through the use of the PPI network and the STRING database (with medium confidence, 0.900). The PPI network diagram was generated and the network topology was analyzed with the CytoNCA plug-in and 191 nodes and 1452 edges larger than the median value were selected. Additional analysis revealed that the core PPI network comprises 46 nodes and 614 edges, with a total of 19 therapeutic targets. These include tyrosine-protein kinase JAK1 (JAK1), focal adhesion kinase 1 (PTK2), tyrosine-protein phosphatase non-receptor type 11 (PTPN11), insulin-like growth factor I receptor (IGF1R), proto-oncogene tyrosine-protein kinase Src (SRC), signal transducer and activator of transcription 3 (STAT3), phosphatidylinositol 4,5-bisphosphate 3-kinase catalytic subunit alphaIsoform (PIK3CA), MAP kinase-activated protein kinase 3 (MAPK3), tyrosine-protein kinase FYN (FYN), signal transducer and activator of transcription 5A (STAT5A), mitogen-activated protein kinase 14 (MAPK14), mitogen-activated protein kinase 1 (MAPK1), Leukokinins (LCK), RAC-alpha serine/threonine-protein kinase (AKT1), tyrosine-protein kinase JAK2 (JAK2), estrogen receptor (ESR1), tyrosine-protein kinase JAK3 (JAK3), epidermal growth factor receptor (EGFR) and transcription factor Jun (JUN). Table 2 and Fig. 3 suggested that these targets could be the fundamental therapeutic targets for breast cancer through LEO treatment.

**Table 2:** Characteristic parameters of target network node of main active ingredients of LEO.

Gene	Betweenness（BC）	Closeness (CC)	Degree (DC)
STAT3	271.2	0.7759	64
SRC	252.5	0.7627	62
MAPK1	143.7	0.7377	58
MAPK3	140.9	0.7377	58
AKT1	85.56	0.6522	42
JUN	71.85	0.6164	34
PIK3CA	66.53	0.6618	46
STAT5A	55.95	0.6522	42
ESR1	39.99	0.6250	36
EGFR	33.96	0.6338	38
PTK2	32.15	0.6000	30
PTPN11	31.47	0.6522	42
JAK2	24.42	0.6250	36
JAK1	23.85	0.6164	38
IGF1R	20.59	0.5844	26
FYN	19.57	0.6164	34
LCK	19.05	0.6338	38
MAPK14	15.01	0.6000	30
JAK3	12.86	0.5921	32

### 3.4 The identification of enriched GO and KEGG pathways offers insights into the characteristics and roles of anticipated targets for treating breast cancer

**Fig. 4:**
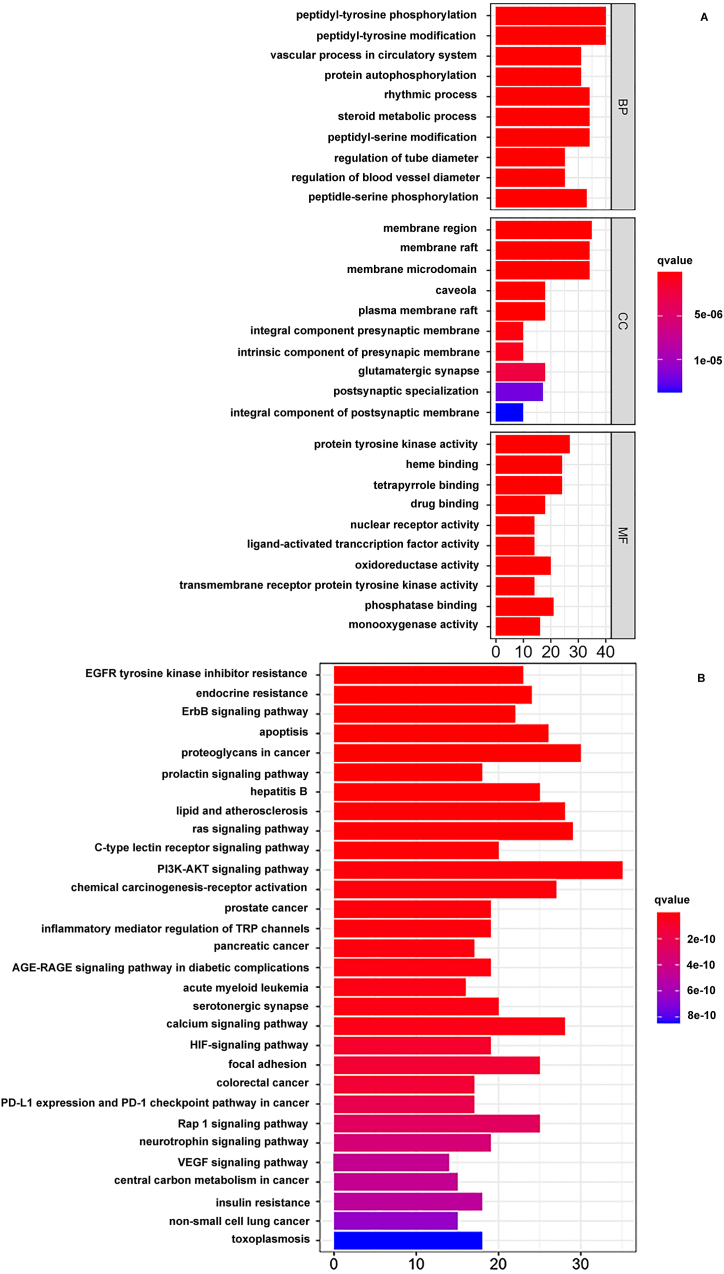
The diagram of GO and KEGG pathway analysis. A: Enrichment analysis of GO function of LEO; B: Enrichment analysis of KEGG pathway of LEO.

The utilization of R software was crucial in conducting a comprehensive assessment and examination of 213 predicted targets, which was culminated in a detailed analysis of GO and KEGG enrichment. When annotating GO functions, we collected 94 entries for cellular component (CC), 217 entries for molecular function (MF) and 2584 entries for biological process (BP).The BP analysis showed that active components of LEO were mainly involved in peptide-tyrosine phosphorylation, peptide-tyrosine modification, peptide-serine phosphorylation, peptide-serine modification, rhythm process and steroid metabolism process; The cell components were mainly distributed in membrane region, membrane raft and cell microdomain; The molecular functions were mainly related to protein tyrosine kinase activity, heme binding, tetrapyrrole binding and phosphatase binding (Fig. 4A). The KEGG enrichment analysis identified 165 signaling pathways and revealed that 213 predictive targets were predominantly associated with phosphoinositide 3-kinase-protein kinase B (PI3K-AKT) signaling pathway, proteoglycans in cancer, the Ras signaling pathway, the calcium signaling pathway, chemical carcinogenesis receptor activation, lipid and atherosclerosis, apoptosis and other signal pathways (Fig. 4B).

### 3.5 Molecular docking of compounds to targets

**Fig. 5:**
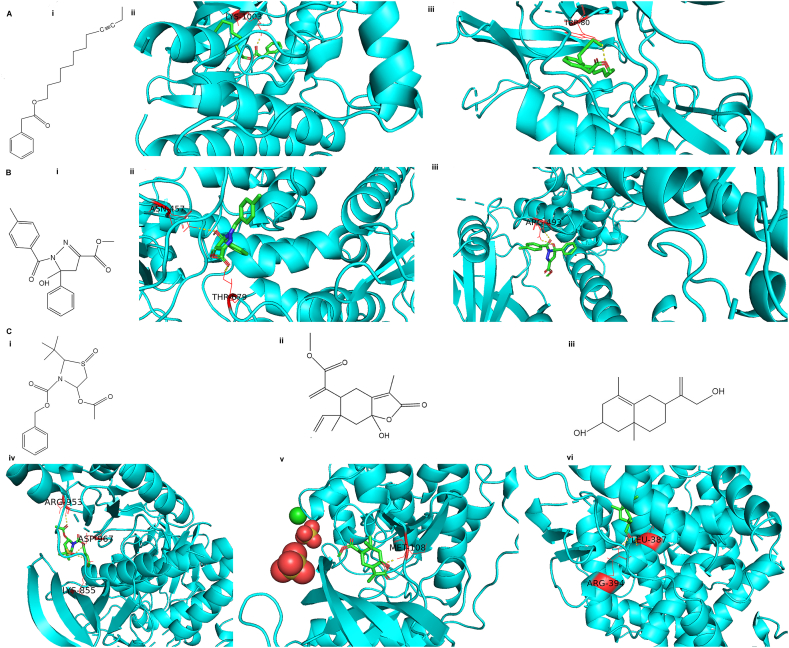
Docking results of five active components of LEO and key targets. A: (i) molecules of phenylacetic acid, dodec-9-ynyl ester (ii) interacted with IGF1R, (iii) interacted with AKT1; B: (i) molecules of 2-pyrazoline-3-carboxylic acid, 5-hydroxy-1-(4-methylbenzoyl)-5-phenyl-, methyl ester, (ii) interacted with PIK3CA, (iii) interacted with STAT5A; C: (i) molecules of 3-thiazolidinecarboxylic acid, 4-(acetyloxy)-2-(1,1-dimethylethyl)-, phenylmethyl ester, 1-oxide, [1R-(1.alpha., 2.beta.,4.beta.)]-, (ii) interacted with JAK3, (iii) molecules of sericealactone (iv) interacted with MAPK1, (v) molecules of bicyclo [4.4.0] dec-5-ene, 1,5-dimethyl-3-hydroxy-8-(1-methylene-2-hydroxyethyl-1)-, (vi) interacted with ESR. The backbones of each protein are depicted in cyan ribbon models. The structure of active components of LEO are shown in spectrum and amino acid residues are in red; Hydrogen bond interactions are shown as yellow dotted lines.

A total of 19 LEO main target proteins and 7 active ingredients were choosed for the molecular docking technique. Table 3 indicated that the sliding score for each interaction is negative, suggesting a favorable between the ligand and the protein. Of all the major targets screened, JAK3, IGF1R, AKT1, PIK3CA, STAT5, MAPK1, and ESR1 present relatively higher negative values for binding energy and amino acid residues involved in interactions with 3-thiazolidinecarboxylic acid, 4-(acetyloxy)-2-(1,1-dimethylethyl)-, phenylmethyl ester, 1-oxide, [1R-(1α, 2,4β)]-, phenylacetic acid, dodec-9-ynyl ester, 2-pyrazoline-3-carboxylic acid, 5-hydroxy-1-(4-methylbenzoyl)-5-phenyl-, methyl ester, sericealactone, and bicyclo [4.4.0]dec-5-ene,1,5-dimethyl-3-hydroxy-8-(1-methylene-2-hydroxyethyl-1)-, respectively. As shown in Fig. 5, the carboxyl oxygen and hydroxy oxygen in ester group of phenylacetic acid, dodec-9-ynyl ester (Fig. 5A i) were predicted to interact by single hydrogen bond with LYS-1003 of IEG1R (Fig. 5A ii) and TRP-80 of AKT1 (Fig. 5A iii), respectively. The carboxyl oxygen in ester group and hydroxyl oxygen of 2-pyrazoline-3-carboxylic acid, 5-hydroxy-1-(4-methylbenzoyl)-5-phenyl-, methyl ester (Fig. 5B i) were predicted to interact by single hydrogen bond with THR-679 and ASN-457 of PIK3CA (Fig. 5B ii) while it's hydroxyl oxygen and carbonyl oxygen were interacted by two hydrogen bonds with APG-493 of STAT5A (Fig. 5B iii). The carboxyl oxygen in ester group of 3-thiazolidinecarboxylic acid, 4-(acetyloxy)-2-(1,1-dimethylethyl)-, phenylmethyl ester (Fig. 5C i) was predicted to interact by single hydrogen bond with ARG-953 of JAK3 and sulfinyl oxygen interacted by double hydrogen bond with ASP-967 and LYS-855 of JAK3 (Fig. 5C iv). The hydroxy oxygen of sericealactone (Fig. 5C ii) interacted by single hydrogen bond with MET108 of MAPK1 (Fig. 5C v). The hydroxy oxygen of 4-(acetyloxy)-2-(1,1-dimethylethyl)-, phenylmethyl ester, 1-oxide, [1R-(1α, 2,4β)]- (Fig. 5C iii), interacted by two hydrogen bond with LEU387 and ARG of ESR1 (Fig. 5C vi).

**Table 3:** The binding energy and interacting amino acid residues of active component with core proteins related to breast cancer.

Ligand	Receptor				Binding energy (kj/mol）	Interaction		Amino acid residues
	Target protein	Gene	UniProt ID	PDB ID		Type	Number	
3-Thiazolidinecarboxylic acid, 4-(acetyloxy)-2-(1,1-dimethylethyl)-, phenylmethyl ester, 1-oxide, [1R-(1.alpha., 2.beta.,4.beta.)]-	Tyrosine-protein kinase JAK1	JAK1	P23458	6SM8	−6.5	hydrogen bond	3	SER-963, GLU966
	Signal transducer and activator of transcription 3	STAT3	P40763	6TLC	−6.9	hydrogen bond	2	GLN-361
	Mitogen-activated protein kinase 14	MAPK14	Q16539	5ETI	−6.0	hydrogen bond	2	ARG-53, ARG-173
	Tyrosine-protein kinase JAK2	JAK2	O60674	6E2Q	−5.8	hydrogen bond	2	LYS-253, ASN-311
	Tyrosine-protein kinase JAK3	JAK3	P52333	4V0G	−7.6	hydrogen bond	3	ARG-953, ASP-967, LYS-855
Phenylacetic acid, dodec-9-ynyl ester	Focal adhesion kinase 1	PTK2	Q05397	6I8Z	−6.3	hydrogen bond	1	CYS-502
	Tyrosine-protein phosphatase non-receptor type 11	PTPN11	Q06124	3B7O	−4.9	hydrogen bond	3	ARG-465, GLN-510
	Insulin-like growth factor I	IGF1R	P05019	3D94	−7.4	hydrogen bond	1	LYS-1003
	Proto-oncogene tyrosine-protein kinase Src	SRC	P12931	2BDF	−6.4	hydrogen bond	1	SER-345
	Tyrosine-protein kinase Lck	LCK	P06239	2OF2	−5.8	hydrogen bond	1	THR-316
	RAC-alpha serine/threonine-protein kinase	AKT1	P31749	3O96	−7.4	hydrogen bond	1	TRP-80
	Epidermal growth factor receptor	EGFR	P00533	3IKA	−6.3	hydrogen bond	1	THR-854
2-Pyrazoline-3-carboxylic acid, 5-hydroxy-1-(4-methylbenzoyl)-5-phenyl-, methyl ester	Phosphatidylinositol 4,5-bisphosphate 3-kinase catalytic subunit alpha isoform	PIK3CA	P42336	6OAC	−7.6	hydrogen bond	2	ASN-457, THR-679
	Signal transducer and activator of transcription 5A	STAT5A	P42229	6MBZ	−7.9	hydrogen bond	2	ARG-493
3-Caren-10-al	MAP kinase-activated protein kinase 3	MAPK3	Q16644	2ZOQ	−6.2	hydrogen bond	1	LYS-224
beta-Terpinyl acetate	Tyrosine-protein kinase Fyn	FYN	P06241	2MRK	−5.1	hydrogen bond	1	TYR-213
Sericealactone	Mitogen-activated protein kinase 1	MAPK1	P28482	6G54	−7.0	hydrogen bond	1	MET-108
	Transcription factor Jun	JUN	P05412	5T01	−6.6	hydrogen bond	2	DC-34, DG-8
Bicyclo [4.4.0]dec-5-ene, 1,5-dimethyl-3-hydroxy-8-(1-methylene-2-hydroxyethyl-1)-	Estrogen receptor	ESR1	P03372	5AAV	−7.9	hydrogen bond	2	LEU-387, ARG-394

### 3.6 LEO demonstrates efficacy in suppressing the proliferation and promoting apoptosis in breast cancer cells

**Fig. 6:**
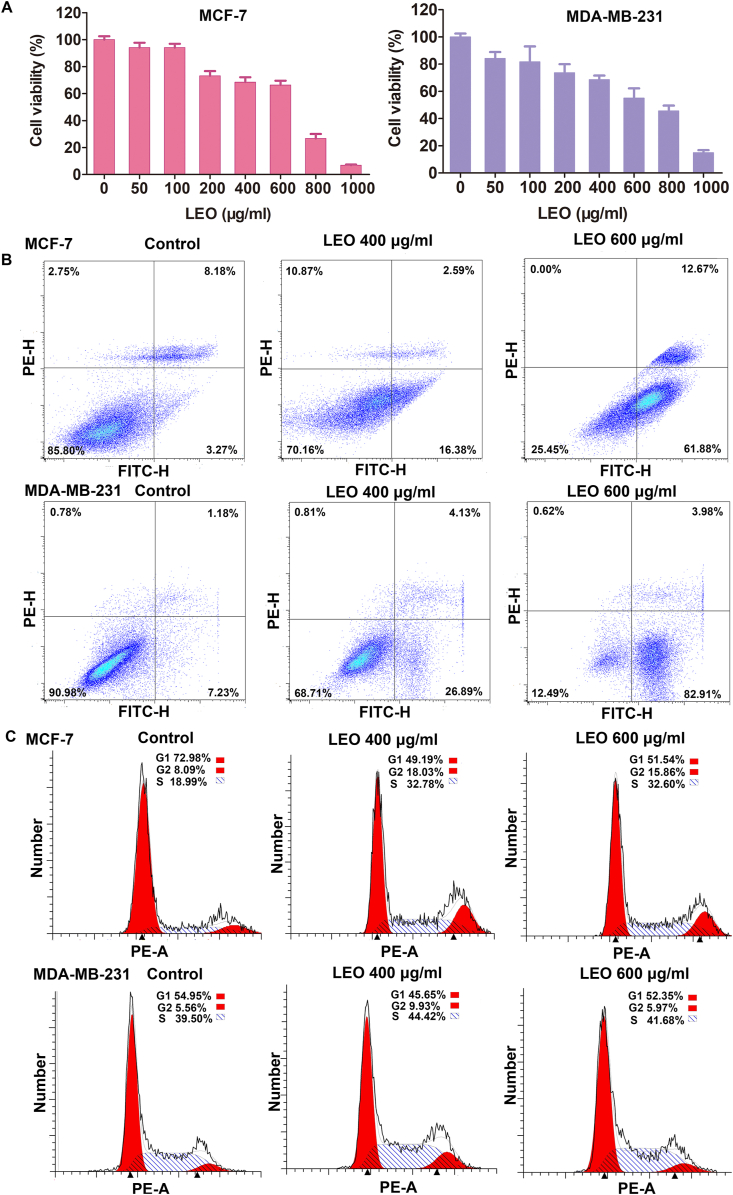
The effect of LEO on the growth of breast cancer cell lines. A: The effect of LEO on the cell viability of breast cancer cell lines. Comparative analysis of each groups indicated statistical significance at p < 0.05 by using one-way ANOVA. B: The effect of LEO on the apoptosis of breast cancer cell lines. C: The effect of LEO on cell cycle arrest.

The effect of LEO on breast carcinoma was observed *in vitro*. To be specific, two distinct breast cancer cell lines, MDA-MB-231 and MCF-7, were employed in the study. A dose-dependent proliferation assay demonstrated the effectiveness of LEO in inhibiting cell proliferation in breast cancer cell lines. The inhibitory effect was more noticeable at higher concentrations of LEO, as depicted in Fig. 6A and Fig. S1. The IC50 values of LEO for MDA-MB-231 cells and MCF-7 cells are respectively 524 μg/mL and 540 μg/mL. By utilizing flow cytometry, an analysis is conducted on how LEO induces apoptosis in these cell lines. The total apoptosis rates of MDA-MB-231 cells were 8.41 %, 31.02 % and 86.88 %, and MCF-7 cells were 11.45 %, 18.97 %, and 74.55 % when exposed with LEO at concentrations of 0, 400 and 600 μg/mL, respectively (Fig. 6B). The results showed that significant differences were detected in the increase of apoptosis following exposure to LEO when compared to the control cells. Furthermore, we employed flow cytometry in conjunction with propidium iodide fluorophores to examine the impact of LEO on cell cycle arrest. There are no significant differences in the percentage of cells in the G1, G2, and S phases treated with LEO at concentrations of 400 μg/mL and 600 μg/mL compared to the untreated cells (Fig. 6C).The results suggested that the LEO induced reduction of the proliferation of MCF-7 and MBA-MD-231 cells by apoptosis but not cell cycle arrest.

### 3.7 The inhibitory effect of LEO on breast cancer could be attributed to its interaction with the PI3K-AKT signaling pathway

**Fig. 7:**
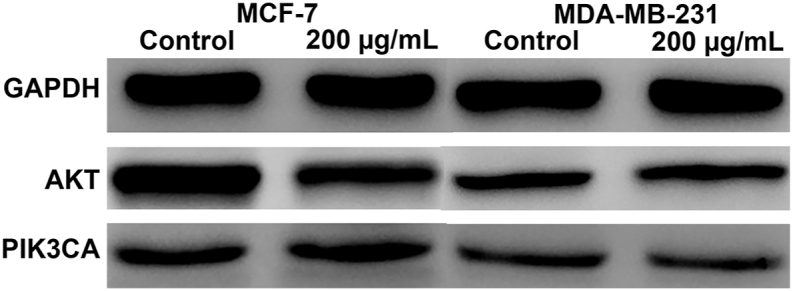
Western blot analysis of AKT1 and PIK3CA in breast cancer cell lines. The protein level of AKT1 and PIK3CA were detected by western blotting. GAPDH was used as internal controls. The results illustrated are from a single experiment and are representative of three separate experiments.

The employment of Western blot analysis examined the effect of LEO on the expression of AKT1 and PIK3CA.The results have provided clarification that LEO caused a reduction in AKT1 expression and an elevation in PIK3CA expression in MCF-7 and MDA-MB-231 cells (Fig. 7 and Fig. S2). The molecular docking study implying that LEO possesses the capability to initiate apoptosis in breast cancer cells by mediating the PI3K-AKT pathway.

## 4 Discussion

The medicinal properties of essential oils, extracted from aromatic plants, have been acknowledged for an extended period of time. Among them, the LEO is the most commonly used one and is generally characterized as “safe” [14]. Based on numerous reports, it appears that the lipophilic nature of essential oil is associated with its high cellular penetration capacity, enabling it to exert its effects in diverse ways within the cells, including by elevating levels of reactive oxygen species (ROS); inhibiting the AKT signaling pathway, mTOR, MAPK, and other crucial biomolecules along with their corresponding genes; expression of the NF-kB; dephosphorylation of AKT; depolarization of the mitochondrial membrane and modulation of DNA repair mechanisms, and finally resulting in apoptosis [15,16]. In terms of anti-cancer properties of LEO, many researchers demonstrated that LEO have significantly anticancer and anti-proliferative activities against various cancer cells [8,14,16]. Donadu and his colleagues assessed the cytotoxic activity of four lavender essential oils against human epithelial colorectal adenocarcinoma cells in their study. The discovery reveals that LEO components, especially linalool and linalool acetate, have the ability to trigger cell death, indicating that LEO could serve as a therapeutic agent for cancer cell lines [17]. Zhao et al. discovered that LEO suppressed the proliferation of human prostate cancer both *in vitro* and *in vivo* [18]. LEO has proven possess the ability to reduce the viability of human cervical cancer cells (HeLa) [14]. However, only a small amount of literature has discussed the anti-breast cancer effects and molecular mechanisms of LEO. According to Boukhatem and Justus's report, Lavender x intermedia essential oils exhibit significant anti-cancer efficacy against the breast cancer cell lines [4,19]. During our research, we stumbled upon the fact that LEO can reduce the survival of cells and trigger programmed cell death in breast cancer cells. Network pharmacology analysis demonstrated that significant amount of “drug-disease” common targets were involved in PI3K-AKT pathway; phenylacetic acid, dodec-9-ynyl ester and 2-Pyrazoline-3-carboxylic acid, 5-hydroxy-1-(4-methylbenzoyl)-5-phenyl-, methyl ester from LEO directly interacted with AKT1 and PIK3CA; western blotting results further confirmed the decrease in AKT1 level and increase in PIK3CA level after treatment with LEO in both breast cancer cell lines. The results indicated that LEO triggers apoptosis in breast cancer cells *in vitro* by manipulating the PI3K-AKT pathway. Although there have been several papers claiming that LEO can trigger apoptosis in breast cancer, this study is the first to elucidate its mechanism of action in terms of PI3K-AKT signaling.

The majority of breast cancers are found to have an active PI3K-AKT pathway, which was previously been linked to numerous tumorigenic functions, such as proliferation, apoptosis, survival, migration and invasion [20]. Genetic mutations and the dysregulation of cell signaling pathways, particularly the PI3K-AKT signaling pathway, exert a major function in initiation and progression of breast cancer, with the latter being the most frequently upregulated pathway in the disease according to previous research [21]. AKT, also known as protein kinase B (PKB), is a type of serine/threonine kinase that has been identified as a pro-survival protein in a PI3K-dependent manner [22]. AKT assumes a crucial function in cell survival by interacting negatively with proteins associated with apoptosis, cell proliferation, cell growth, cell motility and invasion [23]. The gene PIK3CA, which is responsible for producing the PI3K enzyme, plays a crucial role in regulating cell survival, proliferation and metabolism through a cascading positive regulatory mechanism [24]. According to reports, PIK3CA can serve as unfavorable prognostic biomarkers and could be considered for targeted therapy for triple negative breast cancer (TNBC) patients with a poor prognosis [25]. Recently, numerous studies suggest that natural products that target PI3K-AKT have the potential on breast cancer treatments [26]. Besides, except PIK3CA and AKT, we also found IGF1R, MAPK1, JAK3, STAT5A and ESR1 have a relatively higher binding affinity with LEO components. IGF1R is a *trans*-membrane tyrosine kinase receptor and frequently up-regulated in breast cancer [27]. The interaction between the molecules IGF-I and IGF-II, along with the receptor IGF1R, triggers powerful signals that promote cell growth and prevent cell death via the pathways PI3K/AKT and RAS/MAPK [28]. It suggests that in luminal breast cancer with a PIK3CA mutation, MAPK1/3 phosphorylation may play a critical role as a downstream effector worth targeting [29]. The main component of the JAK/STAT signaling pathway are the JAK and STAT proteins, and the alteration of these proteins results increased growth and spread of breast cancer cells [30]. Phosphorylation of STAT5A significantly contributes to the transformation of cancer cells and inhibits apoptosis through up-regulation of the anti-apoptotic protein Bcl-xL [31]. Estrogen receptor alpha (ESR1) and its ligand estradiol (ESR2) is critical for growth of about 70 % of breast cancers [32]. Therefore, the development of effective therapies targeting these genes are under evaluation for their ability to suppress tumor growth and metastasis [30,33]. Despite the many agents have been in clinical use for decades, the new classes of anti-breast cancer drugs targeting above genes should have being developed and our findings will offer novel evidence supporting the utilization of LEO in the management of breast cancer. However, there are many shortcomings presented in our study. Due to the limitation of fund for research, the apoptotic role of LEO and its machanisim could'nt validate *in vivo*; lavendar flower is expensive and sufficient amount of LEO hard to collect for animals treatment; many core targets and involving pathways except AKT1 and PIK3CA needs to analysis. Despite the need for additional research to uncover the underlying mechanisms, our study will make contributions to breast cancer prevention and the development of novel treatment drugs.

## 5 Conclusion

The results of the study suggest that LEO can induce apoptosis in breast cancer cells by mediating the PI3K/AKT signaling pathway. To our knowledge, this study is unique in that it takes a network pharmacology-based approach to investigate the effects of LEO on breast cancer prevention. Our research data offers fresh insights into the therapeutic mechanisms of LEO and presents a viable option for multi-targeted treatment of breast cancer.

## Additional information

Supplementary content related to this article has been published online at [URL].

## Data availability statement

Data will be made available on request.

## CRediT authorship contribution statement

**Guzhalinuer Maitisha:** Writing – review & editing, Writing – original draft, Project administration, Methodology, Investigation, Conceptualization. **Junhao Zhou:** Validation, Methodology, Investigation, Data curation, Conceptualization. **Yan Zhao:** Software, Methodology, Investigation. **Shu xia Han:** Visualization, Formal analysis, Data curation. **Youyun Zhao:** Writing – review & editing, Visualization, Supervision, Resources, Conceptualization. **Ablikim Abliz:** Writing – review & editing, Writing – original draft, Investigation, Funding acquisition, Data curation. **Guangzhong Liu:** Software, Resources, Formal analysis, Data curation.

## Declaration of competing interest

The authors declare that they have no known competing financial interests or personal relationships that could have appeared to influence the work reported in this paper.

## Figures and Tables

**Figure:**
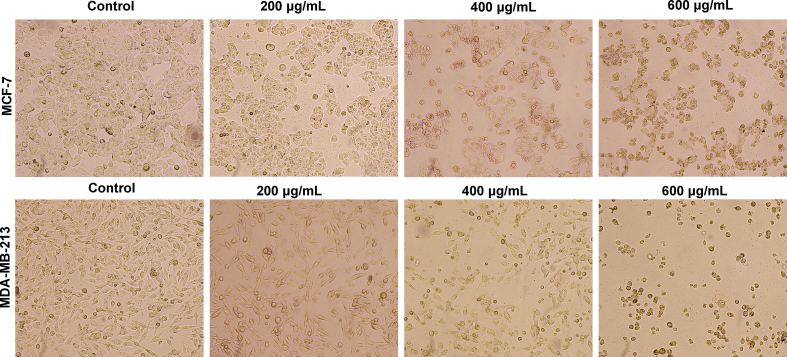
Fig. S1. The dose-dependent effects of LEO on morphology of breast cancer cells（24h, × 100）.

**Figure:**
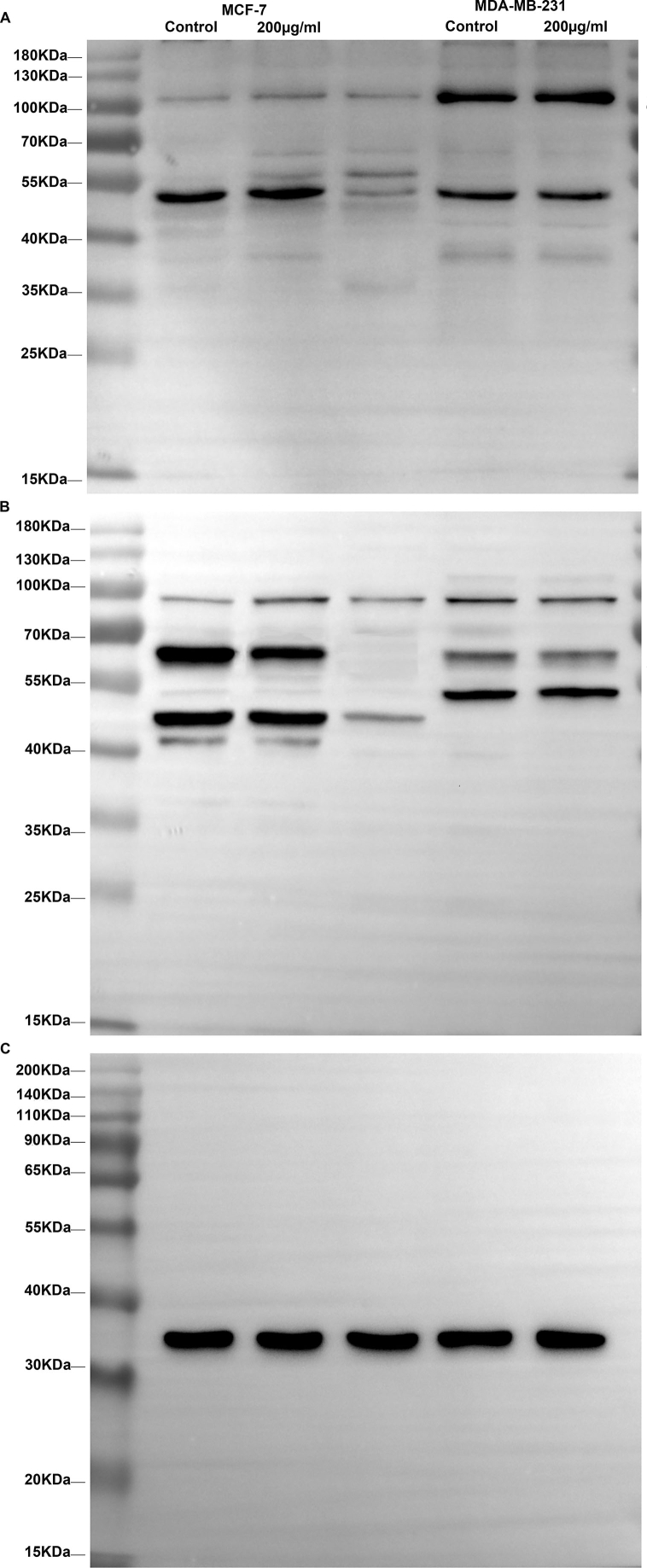
Fig. S2. The original blot of western blot analysis. A: PIK3CA B: AKT1 C: GAPDH.
