# Supplementary material for: Network pharmacology-based approach to investigate the molecular targets of essential oil obtained from lavender for treating breast cancer
Source: Heliyon. 2023 Nov 8;9(11):e21759. doi: 10.1016/j.heliyon.2023.e21759 (PMC10681924; doi:10.1016/j.heliyon.2023.e21759)
Supplement: Multimedia component 1 [file mmc1.docx]

**Table S1 The LEO compounds from DB database**

| **Time** | **CID No** | **Name** | **Formula** | **Canonical SMILES** | **MW** | **Lipinski（DL）** | **GI absorption（OB）** | **P-gp substrate** | **BBB** |
| --- | --- | --- | --- | --- | --- | --- | --- | --- | --- |
| 14.61 | [562380](https://pubchem.ncbi.nlm.nih.gov/compound/562380) | 1,4-Methano-1H-cyclopenta[d]pyridazine, 4,4a,5,7a-tetrahydro-8,8-dimethyl-, (1.alpha.,4.alpha.,4a.alpha.,7a. alpha.)- | C_10_H_14_N_2_ | CC1(C2C3CC=CC3C1N=N2)C | 162 | Yes | High | No | Yes |
|  | [104660](https://pubchem.ncbi.nlm.nih.gov/compound/104660) | 1,5-Decadiyne | C_10_H_14_ | CCCCC#CCCC#C | 134 | Yes; 1 violation | Low | No | Yes |
|  | [576686](https://pubchem.ncbi.nlm.nih.gov/compound/576686) | 2-Pyrazoline-3-carboxylic acid, 5-hydroxy-1-(4-methylbenzoyl)-5-phenyl-, methyl ester | C_19_H_18_N_2_O_4_ | COC(=O)C1=NN(C(=O)C2=CC=C(C)C=C2)C(O)(C1)C1=CC=CC=C1 | 338 | Yes; 0 violation | High | No | No |
|  | [562017](https://pubchem.ncbi.nlm.nih.gov/compound/562017) | 2-Methyl-3,5-dodecadiyne | C_13_H_20_ | CCCCCCC#CC#CC(C)C | 176 | Yes; 1 violation | Low | No | Yes |
| 15.2 | [561847](https://pubchem.ncbi.nlm.nih.gov/compound/561847) | Tricyclo[3.2.1.0(2,4)]octane, 3-methylene- | C_9_H_12_ | C=C1C2C1C1CCC2C1 | 120 | Yes; 0 violation | Low | No | No |
|  | [561846](https://pubchem.ncbi.nlm.nih.gov/compound/561846) | Bicyclo[4.2.0]oct-1-ene, 7-exo-ethenyl- | C_10_H_14_ | C=CC1CC2=CCCCC12 | 134 | Yes; 0 violation | Low | No | Yes |
|  | [564243](https://pubchem.ncbi.nlm.nih.gov/compound/564243) | 8-Methylenebicyclo[4.2.0]oct-4-en-3-one | C_9_H_10_O | C=C1CC2C=CC(=O)CC12 | 134 | Yes; 0 violation | High | No | Yes |
| 15.98 | [7499](https://pubchem.ncbi.nlm.nih.gov/compound/7499) | 4-Vinylcyclohexene | C_8_H_12_ | C=CC1CCC=CC1 | 108 | Yes; 0 violation | Low | No | Yes |
|  | [5362693](https://pubchem.ncbi.nlm.nih.gov/compound/5362693) | 2-Heptene, 1-chloro-, (Z)- | C_7_H_13_Cl | CCCCC=CCCl | 132 | Yes; 0 violation | Low | No | Yes |
|  | [5367344](https://pubchem.ncbi.nlm.nih.gov/compound/5367344) | 3-Decen-1-yne, (Z)- | C_10_H_16_ | CCCCCCC=CC#C | 136 | 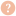Yes; 1 violation | Low | No | Yes |
|  | [7499](https://pubchem.ncbi.nlm.nih.gov/compound/7499) | 4-Vinylcyclohexene | C_8_H_12_ | C=CC1CCC=CC1 | 108 | Yes; 0 violation | Low | No | Yes |
|  | [5365539](https://pubchem.ncbi.nlm.nih.gov/compound/5365539) | 4-Dodecen-2-yne, (Z)- | C_12_H_20_ | CCCCCCCC=CC#CC | 164 | Yes; 1 violation | Low | No | Yes |
| 16.4 | [560987](https://pubchem.ncbi.nlm.nih.gov/compound/560987) | 3-Thiazolidinecarboxylic acid, 4-(acetyloxy)-2-(1,1-dimethylethyl)-, phenylmethyl ester, 1-oxide, [1R-(1.alpha., 2.beta.,4.beta.)]- | C_17_H_23_NO_5_S | CC(=O)OC1CS(=O)CN1C(=O)OCC1=CC=CC=C1 | 353 | Yes; 0 violation | High | No | No |
|  | [522804](https://pubchem.ncbi.nlm.nih.gov/compound/522804) | 4-Benzyloxyphenylacetonitrile | C_15_H_13_NO | C1=CC=C(C=C1)COC2=CC=C(C=C2)CC#N | 223 | Yes; 0 violation | High | No | Yes |
|  | [564243](https://pubchem.ncbi.nlm.nih.gov/compound/564243) | 8-Methylenebicyclo[4.2.0]oct-4-en-3-one | C_9_H_10_O | C=C1CC2C=CC(=O)CC12 | 134 | Yes; 0 violation | High | No | Yes |
|  | [70099](https://pubchem.ncbi.nlm.nih.gov/compound/70099) | 1,7-Octadiyne | C_8_H_10_ | C#CCCCCC#C | 106 | Yes; 0 violation | Low | No | No |
| 17.2 | [557603](https://pubchem.ncbi.nlm.nih.gov/compound/557603) | 3,10-Dioxatricyclo[4.3.1.0(2,4)]dec-7-ene | C_8_H_10_O_2_ | C1C=CC2CC3OC3C1O2 | 138 | Yes; 0 violation | High | No | Yes |
|  | [420988](https://pubchem.ncbi.nlm.nih.gov/compound/6420988) | Bicyclo[2.2.1]heptane, 2-butylidene- | C_11_H_18_ | CCCC=C1CC2CCC1C2 | 150 | Yes; 1 violation | Low | No | Yes |
|  | [556190](https://pubchem.ncbi.nlm.nih.gov/compound/556190) | 1-Dodecen-3-yne | C_12_H_20_ | CCCCCCCCC#CC=C | 164 | Yes; 1 violation | Low | No | Yes |
|  | [544156](https://pubchem.ncbi.nlm.nih.gov/compound/544156) | Acrylic acid 5-methylidene-6-heptenyl ester | C_11_H_16_O_2_ | C=CC(=C)CCCCOC(=O)C=C | 180 | Yes; 0 violation | High | No | Yes |
| 18.2 | [5367427](https://pubchem.ncbi.nlm.nih.gov/compound/5367427) | 3-[(Z)-1-Butenyl]-4-vinylcyclopentene | C_11_H_16_ | CCC=CC1C=CCC1C=C | 148 | Yes; 0 violation | Low | No | Yes |
|  | [12491370](https://pubchem.ncbi.nlm.nih.gov/compound/12491370) | Ectocarpene | C_11_H_16_ | CCC=CC1CC=CCC=C1 | 148 | Yes; 0 violation | Low | No | Yes |
|  | [556420](https://pubchem.ncbi.nlm.nih.gov/compound/556420) | Spiro[cyclopropane-1,6'-[3]oxatricyclo[3.2.1.0(2,4)]octane] | C_9_H_12_O | C1CC11CC2CC1C1OC21 | 136 | Yes; 0 violation | High | No | Yes |
|  | [6673](https://pubchem.ncbi.nlm.nih.gov/compound/6673) | Dicyclopentadiene diepoxide | C_10_H_12_O_2_ | C1C2C3CC4C(C3C1C5C2O5)O4 | 164 | Yes; 0 violation | High | No | Yes |
|  | [74400](https://pubchem.ncbi.nlm.nih.gov/compound/74400) | 1,9-Decadiyne | C_10_H_14_ | C#CCCCCCCC#C | 134 | Yes; 1 violation | Low | No | Yes |
| 18.6 | [560894](https://pubchem.ncbi.nlm.nih.gov/compound/560894) | 1,6-Heptadien-3-yne, 5-methyl- | C_8_H_10_ | CC(C=C)C#CC=C | 106 | Yes; 0 violation | Low | No | No |
|  | [561243](https://pubchem.ncbi.nlm.nih.gov/compound/561243) | 7-Ethyl-1,3,5-cycloheptatriene | C_9_H_12_ | CCC1C=CC=CC=C1 | 120 | Yes; 0 violation | Low | No | Yes |
|  | [144747](https://pubchem.ncbi.nlm.nih.gov/compound/144747) | Bicyclo[3.2.0]hept-2-ene, 7-methylene- | C_8_H_10_ | C=C1CC2CC=CC12 | 106 | Yes; 0 violation | Low | No | No |
|  | [249955570](https://pubchem.ncbi.nlm.nih.gov/substance/249955570) | 7,8-Diazabicyclo[4.2.2]deca-2,4,7,9-tetraen-7-oxide | C_8_H_8_N_2_O | O=N1=NC2C=CC1\C=C/C=C\2 | 148 | Yes; 0 violation | High | Yes | Yes |
| 19.88 | [556274](https://pubchem.ncbi.nlm.nih.gov/compound/556274) | Bicyclo[2.2.1]hept-5-en-2-yl-acetaldehyde | C_9_H_12_O | [H]C(=O)CC1CC2CC1C=C2 | 136 | Yes; 0 violation | High | No | Yes |
|  | [142136](https://pubchem.ncbi.nlm.nih.gov/compound/142136) | 5-exo-Vinyl-5-endo-norbornenol | C_9_H_12_O | C1CC2CC1C=C2O | 136 | Yes; 0 violation | High | No | Yes |
|  | [5367337](https://pubchem.ncbi.nlm.nih.gov/compound/5367337) | 3-Undecen-5-yne, (Z)- | C_11_H_18_ | CCCCCC#CC=CCC | 150 | Yes; 1 violation | Low | No | Yes |
|  | [572048](https://pubchem.ncbi.nlm.nih.gov/compound/572048) | 3-Caren-10-al | C_10_H_14_O | CC1(C2C1CC(=CC2)C=O)C | 150 | Yes; 0 violation | High | No | Yes |
| 21.08 | [39541](https://pubchem.ncbi.nlm.nih.gov/compound/39541) | 2,3-Dimethylenebicyclo[3.2.1]octane | C_10_H_14_ | C=C1CC2CCC(C2)C1=C | 134 | Yes; 0 violation | Low | No | Yes |
|  | [561932](https://pubchem.ncbi.nlm.nih.gov/compound/561932) | Bicyclopentyl-1,1'-diene | C_10_H_14_ | C1CC=C(C1)C2=CCCC2 | 134 | Yes; 0 violation | Low | No | Yes |
|  | [562631](https://pubchem.ncbi.nlm.nih.gov/compound/562631) | Naphthalene, 1,2,3,5,8,8a-hexahydro- | C_10_H_14_ | C1CC=C2CC=CCC2C1 | 134 | Yes; 0 violation | Low | No | Yes |
|  | [562632](https://pubchem.ncbi.nlm.nih.gov/compound/562632) | Cyclohexane, 1,3-butadienylidene- | C_10_H_14_ | C=CC=C=C1CCCCC1 | 134 | Yes; 0 violation | Low | No | Yes |
| 23.94 | [439250](https://pubchem.ncbi.nlm.nih.gov/compound/439250) | (-)-Limonene | C_10_H_16_ | CC1=CCC(CC1)C(=C)C | 136 | Yes; 0 violation | Low | No | Yes |
|  | [549306](https://pubchem.ncbi.nlm.nih.gov/compound/549306) | 1,5-Cyclooctadiene, 3,4-dimethyl- | C_10_H_16_ | CC1C=CCCC=CC1C | 136 | Yes; 0 violation | Low | No | Yes |
|  | [22311](https://pubchem.ncbi.nlm.nih.gov/compound/22311) | Limonene | C_10_H_16_ | CC1=CCC(CC1)C(=C)C | 136 | Yes; 0 violation | Low | No | Yes |
|  | [5365761](https://pubchem.ncbi.nlm.nih.gov/compound/5365761) | 1,6-Dimethylcycloocta-1,5-diene | C_10_H_16_ | CC1=CCCC=C(CC1)C | 136 | Yes; 0 violation | Low | No | Yes |
|  | [88693](https://pubchem.ncbi.nlm.nih.gov/compound/88693) | beta-Terpinyl acetate | C_12_H_20_O_2_ | CC(=C)C1CCC(CC1)(C)OC(=O)C | 196 | Yes; 0 violation | High | No | Yes |
| 25.41 | [5367427](https://pubchem.ncbi.nlm.nih.gov/compound/5367427) | 3-[(Z)-1-Butenyl]-4-vinylcyclopentene | C_11_H_16_ | CCC=CC1C=CCC1C=C | 148 | Yes; 0 violation | Low | No | Yes |
|  | [104660](https://pubchem.ncbi.nlm.nih.gov/compound/104660) | 1,5-Decadiyne | C_10_H_14_ | CCCCC#CCCC#C | 134 | Yes; 0 violation | Low | No | Yes |
|  | [556199](https://pubchem.ncbi.nlm.nih.gov/compound/556199) | Spiro[2.9]dodeca-7,11-diene | C_12_H_18_ | C1CC=CCCC=CC2(C1)CC2 | 162 | Yes; 0 violation | Low | No | Yes |
|  | [562016](https://pubchem.ncbi.nlm.nih.gov/compound/562016) | Cyclopropane, 1-(2-methylene-3-butenyl)-1-(1-methylenepropyl)- | C_12_H_18_ | CCC(=C)C1(CC1)CC(=C)C=C | 162 | Yes; 0 violation | Low | No | Yes |
|  | [6440990](https://pubchem.ncbi.nlm.nih.gov/compound/6440990) | 1-(1-Butenyl)-2,5-cycloheptadiene | C_11_H_16_ | CC/C=C/[C@H]1CC=CCC=C1 | 148 | Yes; 0 violation | Low | No | Yes |
| 26.9 | [5367427](https://pubchem.ncbi.nlm.nih.gov/compound/5367427) | 3-[(Z)-1-Butenyl]-4-vinylcyclopentene | C_11_H_16_ | CCC=CC1C=CCC1C=C | 148 | Yes; 0 violation | Low | No | Yes |
|  | [6440990](https://pubchem.ncbi.nlm.nih.gov/compound/6440990) | 1-(1-Butenyl)-2,5-cycloheptadiene | C_11_H_16_ | CC/C=C/[C@H]1CC=CCC=C1 | 148 | Yes; 0 violation | Low | No | Yes |
|  | [569167](https://pubchem.ncbi.nlm.nih.gov/compound/569167) | 6,6-Dimethyl-2-vinylidenebicyclo[3.1.1]heptane | C_11_H_16_ | CC1(C)C2CC1C(CC2)=C=C | 148 | Yes; 0 violation | Low | No | Yes |
|  | [562618](https://pubchem.ncbi.nlm.nih.gov/compound/562618) | 1,3-Cyclopentadiene, 5-(1,3-dimethylbutylidene)- | C_11_H_16_ | CC(C)CC(=C1C=CC=C1)C | 148 | Yes; 0 violation | Low | No | Yes |
| 28.08 | [70099](https://pubchem.ncbi.nlm.nih.gov/compound/70099) | 1,7-Octadiyne | C_8_H_10_ | C#CCCCCC#C | 106 | Yes; 0 violation | Low | No | Yes |
|  | [144747](https://pubchem.ncbi.nlm.nih.gov/compound/144747) | Bicyclo[3.2.0]hept-2-ene, 7-methylene- | C_8_H_10_ | C=C1CC2CC=CC12 | 106 | Yes; 0 violation | Low | No | No |
|  | [561486](https://pubchem.ncbi.nlm.nih.gov/compound/561486) | Tricyclo[4.2.1.0(2,5)]non-7-en-3-one | C_9_H_10_O | CN1CC1C1=CC=CC=C1 | 134 | Yes; 0 violation | Low | No | No |
| 29.96 | [61275](https://pubchem.ncbi.nlm.nih.gov/compound/61275) | Nerol oxide/2H-Pyran, 3,6-dihydro-4-methyl-2-(2-methyl-1-propenyl)- | C_10_H_16_O | CC1=CCOC(C1)C=C(C)C | 152 | Yes; 0 violation | High | No | Yes |
|  | [561486](https://pubchem.ncbi.nlm.nih.gov/compound/561486) | Tricyclo[4.2.1.0(2,5)]non-7-en-3-one | C_9_H_10_O | CN1CC1C1=CC=CC=C1 | 134 | Yes; 0 violation | Low | No | No |
|  | [74400](https://pubchem.ncbi.nlm.nih.gov/compound/74400) | 1,9-Decadiyne | C_10_H_14_ | C#CCCCCCCC#C | 134 | Yes; 1 violation | Low | No | Yes |
|  | [10819](https://pubchem.ncbi.nlm.nih.gov/compound/10819) | Perillyl alcohol/1-Cyclohexene-1-methanol, 4-(1-methylethenyl)- | C_10_H_16_O | CC(=C)C1CCC(=CC1)CO | 152 | Yes; 0 violation | High | No | Yes |
|  | [561502](https://pubchem.ncbi.nlm.nih.gov/compound/561502) | Phenylacetic acid, dodec-9-ynyl ester | C_20_H_28_O_2_ | CCC#CCCCCCCCCOC(=O)CC1=CC=CC=C1 | 300 | Yes; 1 violation | High | No | Yes |
| 34.5 | [562618](https://pubchem.ncbi.nlm.nih.gov/compound/562618) | 1,3-Cyclopentadiene, 5-(1,3-dimethylbutylidene)- | C_11_H_16_ | CC(C)CC(=C1C=CC=C1)C | 148 | Yes; 0 violation | Low | No | Yes |
|  | [20066](https://pubchem.ncbi.nlm.nih.gov/compound/20066) | 2,8-Decadiyne | C_10_H_14_ | CC#CCCCCC#CC | 134 | Yes; 0 violation | Low | No | Yes |
|  | [561486](https://pubchem.ncbi.nlm.nih.gov/compound/561486) | Tricyclo[4.2.1.0(2,5)]non-7-en-3-one | C_9_H_10_O | O=C1CC2C1C1CC2C=C1 | 134 | Yes; 0 violation | High | No | Yes |
|  | [70099](https://pubchem.ncbi.nlm.nih.gov/compound/70099) | 1,7-Octadiyne | C_8_H_10_ | C#CCCCCC#C | 106 | Yes; 0 violation | Low | No | No |
| 36.17 | [70099](https://pubchem.ncbi.nlm.nih.gov/compound/70099) | 1,7-Octadiyne | C_8_H_10_ | C#CCCCCC#C | 106 | Yes; 0 violation | Low | No | No |
|  | [244005](https://pubchem.ncbi.nlm.nih.gov/compound/244005) | 2-(2-Methylphenyl)propan-2-ol | C_10_H_14_O | CC1=CC=CC=C1C(C)(C)O | 150 | Yes; 0 violation | High | No | Yes |
|  | [20066](https://pubchem.ncbi.nlm.nih.gov/compound/20066) | 2,8-Decadiyne | C_10_H_14_ | CC#CCCCCC#CC | 134 | Yes; 1 violation | Low | No | Yes |
|  | [570471](https://pubchem.ncbi.nlm.nih.gov/compound/570471) | 1,4-Methanophthalazine, 1,4,4a,7,8,8a-hexahydro-9,9-dimethyl-, (1.alpha.,4.alpha.,4a.alpha.,8a.alpha.)- | C_11_H_16_N_2_ | CC1(C)C2N=NC1C1C=CCCC21 | 176 | Yes; 0 violation | High | No | Yes |
| 41.5 | [589284](https://pubchem.ncbi.nlm.nih.gov/compound/589284) | 1,8-Nonadien-3-yne, 2,8-dimethyl-7-methylene- | C_12_H_16_ | CC(=C)C#CCCC(=C)C(=C)C | 160 | Yes; 0 violation | Low | No | Yes |
|  | [561774](https://pubchem.ncbi.nlm.nih.gov/compound/561774) | Acetic acid, isocyano-, phenylmethyl ester | C_10_H_9_NO_2_ | [C-]#[N+]CC(=O)OCC1=CC=CC=C1 | 175 | Yes; 0 violation | High | No | Yes |
|  | [5368337](https://pubchem.ncbi.nlm.nih.gov/compound/5368337) | 6-((1E)-1,3-Butadienyl)-1,4-cycloheptadiene | C_11_H_14_ | C=CC=CC1CC=CCC=C1 | 146 | Yes; 0 violation | Low | No | Yes |
|  | [5368337](https://pubchem.ncbi.nlm.nih.gov/compound/5368337) | 6-((1E)-1,3-Butadienyl)-1,4-cycloheptadiene | C_11_H_14_ | C=C/C=C/C1CC=CCC=C1 | 146 | Yes; 0 violation | Low | No | Yes |
| 48.5 | [555304](https://pubchem.ncbi.nlm.nih.gov/compound/555304) | alpha.-Phenethyl cyanide, 2-methoxy-6-nitro- | C_10_H_10_N_2_O_3_ | COC1=C(C(C)C#N)C(=CC=C1)N(=O)=O | 206 | Yes; 0 violation | High | No | Yes |
|  | [535386](https://pubchem.ncbi.nlm.nih.gov/compound/535386) | Bicyclo[4.4.0]dec-5-ene, 1,5-dimethyl-3-hydroxy-8-(1-methylene-2-hydroxyethyl-1- | C_15_H_24_O_2_ | CC1=C2CC(CCC2(C)CC(O)C1)C(=C)CO | 236 | Yes; 0 violation | High | No | Yes |
|  | [543063](https://pubchem.ncbi.nlm.nih.gov/compound/543063) | Sericealactone（5-Benzofuranacetic acid, 2,4,5,6,7,7a-hexahydro-7a-hydroxy-3,6-dimethyl-.alpha.-methylene-2-oxo-6-vinyl-, methyl ester ） | C_16_H_20_O_5_ | CC1=C2CC(C(CC2(OC1=O)O)(C)C=C)C(=C)C(=O)OC | 292 | Yes; 0 violation | High | No | Yes |
|  | [613267](https://pubchem.ncbi.nlm.nih.gov/compound/613267) | Phenmethylcynide, .alpha.,.alpha.-dimethyl-2-methoxy-6-nitro- | C_11_H_12_N_2_O_3_ | CC1(C)C#[N]OC2=C1C(=CC=C2)N(=O)=O | 220 | Yes; 0 violation | High | No | No |
