# Supplementary material for: Network pharmacology-based approach to investigate the molecular targets of essential oil obtained from lavender for treating breast cancer
Source: Heliyon. 2023 Nov 8;9(11):e21759. doi: 10.1016/j.heliyon.2023.e21759 (PMC10681924; doi:10.1016/j.heliyon.2023.e21759)
Supplement: Multimedia component 2 [file mmc2.docx]

**Table S2 Drug targets of active compounds**

| **No** | **Mol ID** | **Mol Name** | **Symbol** |
| --- | --- | --- | --- |
| 1 | 562380 | 1,4-Methano-1H-cyclopenta[d]pyridazine, 4,4a,5,7a-tetrahydro-8,8-dimethyl-, (1.alpha.,4.alpha.,4a.alpha.,7a. alpha.)- | CA1 |
| 2 | 562380 | 1,4-Methano-1H-cyclopenta[d]pyridazine, 4,4a,5,7a-tetrahydro-8,8-dimethyl-, (1.alpha.,4.alpha.,4a.alpha.,7a. alpha.)- | CA9 |
| 3 | 562380 | 1,4-Methano-1H-cyclopenta[d]pyridazine, 4,4a,5,7a-tetrahydro-8,8-dimethyl-, (1.alpha.,4.alpha.,4a.alpha.,7a. alpha.)- | MIF |
| 4 | 576686 | 2-Pyrazoline-3-carboxylic acid, 5-hydroxy-1-(4-methylbenzoyl)-5-phenyl-, methyl ester | ADORA1 |
| 5 | 576686 | 2-Pyrazoline-3-carboxylic acid, 5-hydroxy-1-(4-methylbenzoyl)-5-phenyl-, methyl ester | AGTR1 |
| 6 | 576686 | 2-Pyrazoline-3-carboxylic acid, 5-hydroxy-1-(4-methylbenzoyl)-5-phenyl-, methyl ester | ALOX15 |
| 7 | 576686 | 2-Pyrazoline-3-carboxylic acid, 5-hydroxy-1-(4-methylbenzoyl)-5-phenyl-, methyl ester | APP |
| 8 | 576686 | 2-Pyrazoline-3-carboxylic acid, 5-hydroxy-1-(4-methylbenzoyl)-5-phenyl-, methyl ester | BDKRB1 |
| 9 | 576686 | 2-Pyrazoline-3-carboxylic acid, 5-hydroxy-1-(4-methylbenzoyl)-5-phenyl-, methyl ester | BRAF |
| 10 | 576686 | 2-Pyrazoline-3-carboxylic acid, 5-hydroxy-1-(4-methylbenzoyl)-5-phenyl-, methyl ester | BRS3 |
| 11 | 576686 | 2-Pyrazoline-3-carboxylic acid, 5-hydroxy-1-(4-methylbenzoyl)-5-phenyl-, methyl ester | CASR |
| 12 | 576686 | 2-Pyrazoline-3-carboxylic acid, 5-hydroxy-1-(4-methylbenzoyl)-5-phenyl-, methyl ester | CCKAR |
| 13 | 576686 | 2-Pyrazoline-3-carboxylic acid, 5-hydroxy-1-(4-methylbenzoyl)-5-phenyl-, methyl ester | CCKBR |
| 14 | 576686 | 2-Pyrazoline-3-carboxylic acid, 5-hydroxy-1-(4-methylbenzoyl)-5-phenyl-, methyl ester | CDK2 |
| 15 | 576686 | 2-Pyrazoline-3-carboxylic acid, 5-hydroxy-1-(4-methylbenzoyl)-5-phenyl-, methyl ester | CTSG |
| 16 | 576686 | 2-Pyrazoline-3-carboxylic acid, 5-hydroxy-1-(4-methylbenzoyl)-5-phenyl-, methyl ester | CXCR2 |
| 17 | 576686 | 2-Pyrazoline-3-carboxylic acid, 5-hydroxy-1-(4-methylbenzoyl)-5-phenyl-, methyl ester | EDNRA |
| 18 | 576686 | 2-Pyrazoline-3-carboxylic acid, 5-hydroxy-1-(4-methylbenzoyl)-5-phenyl-, methyl ester | FKBP1A |
| 19 | 576686 | 2-Pyrazoline-3-carboxylic acid, 5-hydroxy-1-(4-methylbenzoyl)-5-phenyl-, methyl ester | MTOR |
| 20 | 576686 | 2-Pyrazoline-3-carboxylic acid, 5-hydroxy-1-(4-methylbenzoyl)-5-phenyl-, methyl ester | PIK3CA |
| 21 | 576686 | 2-Pyrazoline-3-carboxylic acid, 5-hydroxy-1-(4-methylbenzoyl)-5-phenyl-, methyl ester | STAT5A |
| 22 | 576686 | 2-Pyrazoline-3-carboxylic acid, 5-hydroxy-1-(4-methylbenzoyl)-5-phenyl-, methyl ester | SYK |
| 23 | 560987 | 3-Thiazolidinecarboxylic acid, 4-(acetyloxy)-2-(1,1-dimethylethyl)-, phenylmethyl ester, 1-oxide, [1R-(1.alpha., 2.beta.,4.beta.)]- | CAPN1 |
| 24 | 560987 | 3-Thiazolidinecarboxylic acid, 4-(acetyloxy)-2-(1,1-dimethylethyl)-, phenylmethyl ester, 1-oxide, [1R-(1.alpha., 2.beta.,4.beta.)]- | CAPN2 |
| 25 | 560987 | 3-Thiazolidinecarboxylic acid, 4-(acetyloxy)-2-(1,1-dimethylethyl)-, phenylmethyl ester, 1-oxide, [1R-(1.alpha., 2.beta.,4.beta.)]- | CHRM1 |
| 26 | 560987 | 3-Thiazolidinecarboxylic acid, 4-(acetyloxy)-2-(1,1-dimethylethyl)-, phenylmethyl ester, 1-oxide, [1R-(1.alpha., 2.beta.,4.beta.)]- | CHRM2 |
| 27 | 560987 | 3-Thiazolidinecarboxylic acid, 4-(acetyloxy)-2-(1,1-dimethylethyl)-, phenylmethyl ester, 1-oxide, [1R-(1.alpha., 2.beta.,4.beta.)]- | CSF1R |
| 28 | 560987 | 3-Thiazolidinecarboxylic acid, 4-(acetyloxy)-2-(1,1-dimethylethyl)-, phenylmethyl ester, 1-oxide, [1R-(1.alpha., 2.beta.,4.beta.)]- | DRD2 |
| 29 | 560987 | 3-Thiazolidinecarboxylic acid, 4-(acetyloxy)-2-(1,1-dimethylethyl)-, phenylmethyl ester, 1-oxide, [1R-(1.alpha., 2.beta.,4.beta.)]- | DRD4 |
| 30 | 560987 | 3-Thiazolidinecarboxylic acid, 4-(acetyloxy)-2-(1,1-dimethylethyl)-, phenylmethyl ester, 1-oxide, [1R-(1.alpha., 2.beta.,4.beta.)]- | FAAH |
| 31 | 560987 | 3-Thiazolidinecarboxylic acid, 4-(acetyloxy)-2-(1,1-dimethylethyl)-, phenylmethyl ester, 1-oxide, [1R-(1.alpha., 2.beta.,4.beta.)]- | GSK3B |
| 32 | 560987 | 3-Thiazolidinecarboxylic acid, 4-(acetyloxy)-2-(1,1-dimethylethyl)-, phenylmethyl ester, 1-oxide, [1R-(1.alpha., 2.beta.,4.beta.)]- | HCRTR1 |
| 33 | 560987 | 3-Thiazolidinecarboxylic acid, 4-(acetyloxy)-2-(1,1-dimethylethyl)-, phenylmethyl ester, 1-oxide, [1R-(1.alpha., 2.beta.,4.beta.)]- | HDAC1 |
| 34 | 560987 | 3-Thiazolidinecarboxylic acid, 4-(acetyloxy)-2-(1,1-dimethylethyl)-, phenylmethyl ester, 1-oxide, [1R-(1.alpha., 2.beta.,4.beta.)]- | HDAC6 |
| 35 | 560987 | 3-Thiazolidinecarboxylic acid, 4-(acetyloxy)-2-(1,1-dimethylethyl)-, phenylmethyl ester, 1-oxide, [1R-(1.alpha., 2.beta.,4.beta.)]- | HPGDS |
| 36 | 560987 | 3-Thiazolidinecarboxylic acid, 4-(acetyloxy)-2-(1,1-dimethylethyl)-, phenylmethyl ester, 1-oxide, [1R-(1.alpha., 2.beta.,4.beta.)]- | IDO1 |
| 37 | 560987 | 3-Thiazolidinecarboxylic acid, 4-(acetyloxy)-2-(1,1-dimethylethyl)-, phenylmethyl ester, 1-oxide, [1R-(1.alpha., 2.beta.,4.beta.)]- | JAK1 |
| 38 | 560987 | 3-Thiazolidinecarboxylic acid, 4-(acetyloxy)-2-(1,1-dimethylethyl)-, phenylmethyl ester, 1-oxide, [1R-(1.alpha., 2.beta.,4.beta.)]- | JAK2 |
| 39 | 560987 | 3-Thiazolidinecarboxylic acid, 4-(acetyloxy)-2-(1,1-dimethylethyl)-, phenylmethyl ester, 1-oxide, [1R-(1.alpha., 2.beta.,4.beta.)]- | JAK3 |
| 40 | 560987 | 3-Thiazolidinecarboxylic acid, 4-(acetyloxy)-2-(1,1-dimethylethyl)-, phenylmethyl ester, 1-oxide, [1R-(1.alpha., 2.beta.,4.beta.)]- | KCNA5 |
| 41 | 560987 | 3-Thiazolidinecarboxylic acid, 4-(acetyloxy)-2-(1,1-dimethylethyl)-, phenylmethyl ester, 1-oxide, [1R-(1.alpha., 2.beta.,4.beta.)]- | KCNH2 |
| 42 | 560987 | 3-Thiazolidinecarboxylic acid, 4-(acetyloxy)-2-(1,1-dimethylethyl)-, phenylmethyl ester, 1-oxide, [1R-(1.alpha., 2.beta.,4.beta.)]- | MAPK10 |
| 43 | 560987 | 3-Thiazolidinecarboxylic acid, 4-(acetyloxy)-2-(1,1-dimethylethyl)-, phenylmethyl ester, 1-oxide, [1R-(1.alpha., 2.beta.,4.beta.)]- | MAPK14 |
| 44 | 560987 | 3-Thiazolidinecarboxylic acid, 4-(acetyloxy)-2-(1,1-dimethylethyl)-, phenylmethyl ester, 1-oxide, [1R-(1.alpha., 2.beta.,4.beta.)]- | MDM2 |
| 45 | 560987 | 3-Thiazolidinecarboxylic acid, 4-(acetyloxy)-2-(1,1-dimethylethyl)-, phenylmethyl ester, 1-oxide, [1R-(1.alpha., 2.beta.,4.beta.)]- | NOS1 |
| 46 | 560987 | 3-Thiazolidinecarboxylic acid, 4-(acetyloxy)-2-(1,1-dimethylethyl)-, phenylmethyl ester, 1-oxide, [1R-(1.alpha., 2.beta.,4.beta.)]- | NOS2 |
| 47 | 560987 | 3-Thiazolidinecarboxylic acid, 4-(acetyloxy)-2-(1,1-dimethylethyl)-, phenylmethyl ester, 1-oxide, [1R-(1.alpha., 2.beta.,4.beta.)]- | NOS3 |
| 48 | 560987 | 3-Thiazolidinecarboxylic acid, 4-(acetyloxy)-2-(1,1-dimethylethyl)-, phenylmethyl ester, 1-oxide, [1R-(1.alpha., 2.beta.,4.beta.)]- | NR3C1 |
| 49 | 560987 | 3-Thiazolidinecarboxylic acid, 4-(acetyloxy)-2-(1,1-dimethylethyl)-, phenylmethyl ester, 1-oxide, [1R-(1.alpha., 2.beta.,4.beta.)]- | NR3C2 |
| 50 | 560987 | 3-Thiazolidinecarboxylic acid, 4-(acetyloxy)-2-(1,1-dimethylethyl)-, phenylmethyl ester, 1-oxide, [1R-(1.alpha., 2.beta.,4.beta.)]- | PARP1 |
| 51 | 560987 | 3-Thiazolidinecarboxylic acid, 4-(acetyloxy)-2-(1,1-dimethylethyl)-, phenylmethyl ester, 1-oxide, [1R-(1.alpha., 2.beta.,4.beta.)]- | PDE3A |
| 52 | 560987 | 3-Thiazolidinecarboxylic acid, 4-(acetyloxy)-2-(1,1-dimethylethyl)-, phenylmethyl ester, 1-oxide, [1R-(1.alpha., 2.beta.,4.beta.)]- | PRKCG |
| 53 | 560987 | 3-Thiazolidinecarboxylic acid, 4-(acetyloxy)-2-(1,1-dimethylethyl)-, phenylmethyl ester, 1-oxide, [1R-(1.alpha., 2.beta.,4.beta.)]- | PTGS2 |
| 54 | 560987 | 3-Thiazolidinecarboxylic acid, 4-(acetyloxy)-2-(1,1-dimethylethyl)-, phenylmethyl ester, 1-oxide, [1R-(1.alpha., 2.beta.,4.beta.)]- | RIPK1 |
| 55 | 560987 | 3-Thiazolidinecarboxylic acid, 4-(acetyloxy)-2-(1,1-dimethylethyl)-, phenylmethyl ester, 1-oxide, [1R-(1.alpha., 2.beta.,4.beta.)]- | SCN2A |
| 56 | 560987 | 3-Thiazolidinecarboxylic acid, 4-(acetyloxy)-2-(1,1-dimethylethyl)-, phenylmethyl ester, 1-oxide, [1R-(1.alpha., 2.beta.,4.beta.)]- | SCN3A |
| 57 | 560987 | 3-Thiazolidinecarboxylic acid, 4-(acetyloxy)-2-(1,1-dimethylethyl)-, phenylmethyl ester, 1-oxide, [1R-(1.alpha., 2.beta.,4.beta.)]- | SCN5A |
| 58 | 560987 | 3-Thiazolidinecarboxylic acid, 4-(acetyloxy)-2-(1,1-dimethylethyl)-, phenylmethyl ester, 1-oxide, [1R-(1.alpha., 2.beta.,4.beta.)]- | SCN9A |
| 59 | 560987 | 3-Thiazolidinecarboxylic acid, 4-(acetyloxy)-2-(1,1-dimethylethyl)-, phenylmethyl ester, 1-oxide, [1R-(1.alpha., 2.beta.,4.beta.)]- | SLC6A3 |
| 60 | 560987 | 3-Thiazolidinecarboxylic acid, 4-(acetyloxy)-2-(1,1-dimethylethyl)-, phenylmethyl ester, 1-oxide, [1R-(1.alpha., 2.beta.,4.beta.)]- | STAT3 |
| 61 | 560987 | 3-Thiazolidinecarboxylic acid, 4-(acetyloxy)-2-(1,1-dimethylethyl)-, phenylmethyl ester, 1-oxide, [1R-(1.alpha., 2.beta.,4.beta.)]- | TDO2 |
| 62 | 560987 | 3-Thiazolidinecarboxylic acid, 4-(acetyloxy)-2-(1,1-dimethylethyl)-, phenylmethyl ester, 1-oxide, [1R-(1.alpha., 2.beta.,4.beta.)]- | TNKS2 |
| 63 | 522804 | 4-Benzyloxyphenylacetonitrile | ALOX5 |
| 64 | 522804 | 4-Benzyloxyphenylacetonitrile | CYP2A6 |
| 65 | 522804 | 4-Benzyloxyphenylacetonitrile | CYP2C19 |
| 66 | 522804 | 4-Benzyloxyphenylacetonitrile | GABRA1 |
| 67 | 522804 | 4-Benzyloxyphenylacetonitrile | GABRA5 |
| 68 | 522804 | 4-Benzyloxyphenylacetonitrile | MAOA |
| 69 | 522804 | 4-Benzyloxyphenylacetonitrile | MAOB |
| 70 | 522804 | 4-Benzyloxyphenylacetonitrile | MPO |
| 71 | 522804 | 4-Benzyloxyphenylacetonitrile | PABPC1 |
| 72 | 522804 | 4-Benzyloxyphenylacetonitrile | PIK3CB |
| 73 | 522804 | 4-Benzyloxyphenylacetonitrile | RPS6KB1 |
| 74 | 522804 | 4-Benzyloxyphenylacetonitrile | TBXAS1 |
| 75 | 522804 | 4-Benzyloxyphenylacetonitrile | TNKS |
| 76 | 522804 | 4-Benzyloxyphenylacetonitrile | XPO1 |
| 77 | 557603 | 3,10-Dioxatricyclo[4.3.1.0(2,4)]dec-7-ene | PRKCA |
| 78 | 557603 | 3,10-Dioxatricyclo[4.3.1.0(2,4)]dec-7-ene | PRKCD |
| 79 | [544156](https://pubchem.ncbi.nlm.nih.gov/compound/544156) | Acrylic acid 5-methylidene-6-heptenyl ester | CHRM3 |
| 80 | [544156](https://pubchem.ncbi.nlm.nih.gov/compound/544156) | Acrylic acid 5-methylidene-6-heptenyl ester | CHRM5 |
| 81 | 556420 | Spiro[cyclopropane-1,6'-[3]oxatricyclo[3.2.1.0(2,4)]octane] | CYP19A1 |
| 82 | 6673 | Dicyclopentadiene diepoxide | CYP51A1 |
| 83 | 6673 | Dicyclopentadiene diepoxide | GBA |
| 84 | 6673 | Dicyclopentadiene diepoxide | SHH |
| 85 | [556274](https://pubchem.ncbi.nlm.nih.gov/compound/556274) | Bicyclo[2.2.1]hept-5-en-2-yl-acetaldehyde | CCR5 |
| 86 | [556274](https://pubchem.ncbi.nlm.nih.gov/compound/556274) | Bicyclo[2.2.1]hept-5-en-2-yl-acetaldehyde | CTSD |
| 87 | [556274](https://pubchem.ncbi.nlm.nih.gov/compound/556274) | Bicyclo[2.2.1]hept-5-en-2-yl-acetaldehyde | FABP3 |
| 88 | [556274](https://pubchem.ncbi.nlm.nih.gov/compound/556274) | Bicyclo[2.2.1]hept-5-en-2-yl-acetaldehyde | FABP4 |
| 89 | [556274](https://pubchem.ncbi.nlm.nih.gov/compound/556274) | Bicyclo[2.2.1]hept-5-en-2-yl-acetaldehyde | FABP5 |
| 90 | [556274](https://pubchem.ncbi.nlm.nih.gov/compound/556274) | Bicyclo[2.2.1]hept-5-en-2-yl-acetaldehyde | PPARD |
| 91 | [556274](https://pubchem.ncbi.nlm.nih.gov/compound/556274) | Bicyclo[2.2.1]hept-5-en-2-yl-acetaldehyde | PTGS1 |
| 92 | [556274](https://pubchem.ncbi.nlm.nih.gov/compound/556274) | Bicyclo[2.2.1]hept-5-en-2-yl-acetaldehyde | PTPN6 |
| 93 | [556274](https://pubchem.ncbi.nlm.nih.gov/compound/556274) | Bicyclo[2.2.1]hept-5-en-2-yl-acetaldehyde | SCD |
| 94 | [556274](https://pubchem.ncbi.nlm.nih.gov/compound/556274) | Bicyclo[2.2.1]hept-5-en-2-yl-acetaldehyde | TPO |
| 95 | 572048 | 3-Caren-10-al | HSD11B2 |
| 96 | 572048 | 3-Caren-10-al | HSD17B3 |
| 97 | 572048 | 3-Caren-10-al | MAPK3 |
| 98 | 572048 | 3-Caren-10-al | PRKCH |
| 99 | 572048 | 3-Caren-10-al | PTGES |
| 100 | 572048 | 3-Caren-10-al | PTPN2 |
| 101 | 572048 | 3-Caren-10-al | SERPINA6 |
| 102 | 572048 | 3-Caren-10-al | SRD5A1 |
| 103 | 572048 | 3-Caren-10-al | SRD5A2 |
| 104 | 572048 | 3-Caren-10-al | TRPA1 |
| 105 | 561932 | beta-Terpinyl acetate | CEL |
| 106 | 561932 | beta-Terpinyl acetate | CES2 |
| 107 | 561932 | beta-Terpinyl acetate | CTRB1 |
| 108 | 561932 | beta-Terpinyl acetate | CTRC |
| 109 | 561932 | beta-Terpinyl acetate | CYP1A2 |
| 110 | 561932 | beta-Terpinyl acetate | CYP2C9 |
| 111 | 561932 | beta-Terpinyl acetate | CYP2D6 |
| 112 | 561932 | beta-Terpinyl acetate | CYP3A4 |
| 113 | 561932 | beta-Terpinyl acetate | F2 |
| 114 | 561932 | beta-Terpinyl acetate | FYN |
| 115 | 561932 | beta-Terpinyl acetate | HSD11B1 |
| 116 | 561932 | beta-Terpinyl acetate | HTT |
| 117 | 561932 | beta-Terpinyl acetate | LIPE |
| 118 | 561932 | beta-Terpinyl acetate | PLA2G6 |
| 119 | 561932 | beta-Terpinyl acetate | PLAU |
| 120 | 561932 | beta-Terpinyl acetate | PRKDC |
| 121 | 561932 | beta-Terpinyl acetate | PRSS1 |
| 122 | 561932 | beta-Terpinyl acetate | RAF1 |
| 123 | 561932 | beta-Terpinyl acetate | TOP2A |
| 124 | 61275 | Nerol oxide/2H-Pyran, 3,6-dihydro-4-methyl-2-(2-methyl-1-propenyl)- | CTSB |
| 125 | 61275 | Nerol oxide/2H-Pyran, 3,6-dihydro-4-methyl-2-(2-methyl-1-propenyl)- | CTSK |
| 126 | 61275 | Nerol oxide/2H-Pyran, 3,6-dihydro-4-methyl-2-(2-methyl-1-propenyl)- | CTSL |
| 127 | 61275 | Nerol oxide/2H-Pyran, 3,6-dihydro-4-methyl-2-(2-methyl-1-propenyl)- | NLRP3 |
| 128 | 10819 | Perillyl alcohol/1-Cyclohexene-1-methanol, 4-(1-methylethenyl)- | CYP11B1 |
| 129 | 10819 | Perillyl alcohol/1-Cyclohexene-1-methanol, 4-(1-methylethenyl)- | CYP11B2 |
| 130 | 10819 | Perillyl alcohol/1-Cyclohexene-1-methanol, 4-(1-methylethenyl)- | SQLE |
| 131 | 561502 | Phenylacetic acid, dodec-9-ynyl ester | ABL1 |
| 132 | 561502 | Phenylacetic acid, dodec-9-ynyl ester | ADK |
| 133 | 561502 | Phenylacetic acid, dodec-9-ynyl ester | ADRB1 |
| 134 | 561502 | Phenylacetic acid, dodec-9-ynyl ester | ADRB2 |
| 135 | 561502 | Phenylacetic acid, dodec-9-ynyl ester | AKT1 |
| 136 | 561502 | Phenylacetic acid, dodec-9-ynyl ester | AURKA |
| 137 | 561502 | Phenylacetic acid, dodec-9-ynyl ester | AURKB |
| 138 | 561502 | Phenylacetic acid, dodec-9-ynyl ester | BCL2 |
| 139 | 561502 | Phenylacetic acid, dodec-9-ynyl ester | BCL2L1 |
| 140 | 561502 | Phenylacetic acid, dodec-9-ynyl ester | CNR1 |
| 141 | 561502 | Phenylacetic acid, dodec-9-ynyl ester | CNR2 |
| 142 | 561502 | Phenylacetic acid, dodec-9-ynyl ester | EGFR |
| 143 | 561502 | Phenylacetic acid, dodec-9-ynyl ester | EPHB4 |
| 144 | 561502 | Phenylacetic acid, dodec-9-ynyl ester | ERBB2 |
| 145 | 561502 | Phenylacetic acid, dodec-9-ynyl ester | FLT3 |
| 146 | 561502 | Phenylacetic acid, dodec-9-ynyl ester | FLT4 |
| 147 | 561502 | Phenylacetic acid, dodec-9-ynyl ester | GRIN2B |
| 148 | 561502 | Phenylacetic acid, dodec-9-ynyl ester | GRM5 |
| 149 | 561502 | Phenylacetic acid, dodec-9-ynyl ester | IDH1 |
| 150 | 561502 | Phenylacetic acid, dodec-9-ynyl ester | IGF1R |
| 151 | 561502 | Phenylacetic acid, dodec-9-ynyl ester | INSR |
| 152 | 561502 | Phenylacetic acid, dodec-9-ynyl ester | KDR |
| 153 | 561502 | Phenylacetic acid, dodec-9-ynyl ester | KIT |
| 154 | 561502 | Phenylacetic acid, dodec-9-ynyl ester | LCK |
| 155 | 561502 | Phenylacetic acid, dodec-9-ynyl ester | LSS |
| 156 | 561502 | Phenylacetic acid, dodec-9-ynyl ester | MAPK8 |
| 157 | 561502 | Phenylacetic acid, dodec-9-ynyl ester | MAPK9 |
| 158 | 561502 | Phenylacetic acid, dodec-9-ynyl ester | MCL1 |
| 159 | 561502 | Phenylacetic acid, dodec-9-ynyl ester | NTRK1 |
| 160 | 561502 | Phenylacetic acid, dodec-9-ynyl ester | PDE10A |
| 161 | 561502 | Phenylacetic acid, dodec-9-ynyl ester | PDE4B |
| 162 | 561502 | Phenylacetic acid, dodec-9-ynyl ester | PGR |
| 163 | 561502 | Phenylacetic acid, dodec-9-ynyl ester | PTK2 |
| 164 | 561502 | Phenylacetic acid, dodec-9-ynyl ester | PTPN11 |
| 165 | 561502 | Phenylacetic acid, dodec-9-ynyl ester | SMO |
| 166 | 561502 | Phenylacetic acid, dodec-9-ynyl ester | SRC |
| 167 | 561502 | Phenylacetic acid, dodec-9-ynyl ester | SREBF2 |
| 168 | 561502 | Phenylacetic acid, dodec-9-ynyl ester | TEK |
| 169 | 561502 | Phenylacetic acid, dodec-9-ynyl ester | TRPV1 |
| 170 | 561502 | Phenylacetic acid, dodec-9-ynyl ester | YES1 |
| 171 | 244005 | 2-(2-Methylphenyl)propan-2-ol | TRPM8 |
| 172 | 244005 | 2-(2-Methylphenyl)propan-2-ol | TRPV3 |
| 173 | 555304 | alpha.-Phenethyl cyanide, 2-methoxy-6-nitro- | KAT2B |
| 174 | 535386 | Bicyclo[4.4.0]dec-5-ene, 1,5-dimethyl-3-hydroxy-8-(1-methylene-2-hydroxyethyl-1)- | AR |
| 175 | 535386 | Bicyclo[4.4.0]dec-5-ene, 1,5-dimethyl-3-hydroxy-8-(1-methylene-2-hydroxyethyl-1)- | ESR1 |
| 176 | 535386 | Bicyclo[4.4.0]dec-5-ene, 1,5-dimethyl-3-hydroxy-8-(1-methylene-2-hydroxyethyl-1)- | ESR2 |
| 177 | 535386 | Bicyclo[4.4.0]dec-5-ene, 1,5-dimethyl-3-hydroxy-8-(1-methylene-2-hydroxyethyl-1)- | NPC1L1 |
| 178 | 535386 | Bicyclo[4.4.0]dec-5-ene, 1,5-dimethyl-3-hydroxy-8-(1-methylene-2-hydroxyethyl-1)- | NR1H2 |
| 179 | 535386 | Bicyclo[4.4.0]dec-5-ene, 1,5-dimethyl-3-hydroxy-8-(1-methylene-2-hydroxyethyl-1)- | NR1H3 |
| 180 | 535386 | Bicyclo[4.4.0]dec-5-ene, 1,5-dimethyl-3-hydroxy-8-(1-methylene-2-hydroxyethyl-1)- | PPARA |
| 181 | 535386 | Bicyclo[4.4.0]dec-5-ene, 1,5-dimethyl-3-hydroxy-8-(1-methylene-2-hydroxyethyl-1)- | RORA |
| 182 | 535386 | Bicyclo[4.4.0]dec-5-ene, 1,5-dimethyl-3-hydroxy-8-(1-methylene-2-hydroxyethyl-1)- | RORC |
| 183 | 535386 | Bicyclo[4.4.0]dec-5-ene, 1,5-dimethyl-3-hydroxy-8-(1-methylene-2-hydroxyethyl-1)- | SHBG |
| 184 | 535386 | Bicyclo[4.4.0]dec-5-ene, 1,5-dimethyl-3-hydroxy-8-(1-methylene-2-hydroxyethyl-1)- | TYK2 |
| 185 | 543063 | Sericealactone（5-Benzofuranacetic acid, 2,4,5,6,7,7a-hexahydro-7a-hydroxy-3,6-dimethyl-.alpha.-methylene-2-oxo-6-vinyl-, methyl ester ） | ADORA2A |
| 186 | 543063 | Sericealactone（5-Benzofuranacetic acid, 2,4,5,6,7,7a-hexahydro-7a-hydroxy-3,6-dimethyl-.alpha.-methylene-2-oxo-6-vinyl-, methyl ester ） | ADRA1A |
| 187 | 543063 | Sericealactone（5-Benzofuranacetic acid, 2,4,5,6,7,7a-hexahydro-7a-hydroxy-3,6-dimethyl-.alpha.-methylene-2-oxo-6-vinyl-, methyl ester ） | ADRA1B |
| 188 | 543063 | Sericealactone（5-Benzofuranacetic acid, 2,4,5,6,7,7a-hexahydro-7a-hydroxy-3,6-dimethyl-.alpha.-methylene-2-oxo-6-vinyl-, methyl ester ） | ADRA1D |
| 189 | 543063 | Sericealactone（5-Benzofuranacetic acid, 2,4,5,6,7,7a-hexahydro-7a-hydroxy-3,6-dimethyl-.alpha.-methylene-2-oxo-6-vinyl-, methyl ester ） | ALOX12 |
| 190 | 543063 | Sericealactone（5-Benzofuranacetic acid, 2,4,5,6,7,7a-hexahydro-7a-hydroxy-3,6-dimethyl-.alpha.-methylene-2-oxo-6-vinyl-, methyl ester ） | ATP12A |
| 191 | 543063 | Sericealactone（5-Benzofuranacetic acid, 2,4,5,6,7,7a-hexahydro-7a-hydroxy-3,6-dimethyl-.alpha.-methylene-2-oxo-6-vinyl-, methyl ester ） | BRD4 |
| 192 | 543063 | Sericealactone（5-Benzofuranacetic acid, 2,4,5,6,7,7a-hexahydro-7a-hydroxy-3,6-dimethyl-.alpha.-methylene-2-oxo-6-vinyl-, methyl ester ） | CASP1 |
| 193 | 543063 | Sericealactone（5-Benzofuranacetic acid, 2,4,5,6,7,7a-hexahydro-7a-hydroxy-3,6-dimethyl-.alpha.-methylene-2-oxo-6-vinyl-, methyl ester ） | CASP3 |
| 194 | 543063 | Sericealactone（5-Benzofuranacetic acid, 2,4,5,6,7,7a-hexahydro-7a-hydroxy-3,6-dimethyl-.alpha.-methylene-2-oxo-6-vinyl-, methyl ester ） | CASP6 |
| 195 | 543063 | Sericealactone（5-Benzofuranacetic acid, 2,4,5,6,7,7a-hexahydro-7a-hydroxy-3,6-dimethyl-.alpha.-methylene-2-oxo-6-vinyl-, methyl ester ） | CASP7 |
| 196 | 543063 | Sericealactone（5-Benzofuranacetic acid, 2,4,5,6,7,7a-hexahydro-7a-hydroxy-3,6-dimethyl-.alpha.-methylene-2-oxo-6-vinyl-, methyl ester ） | CASP8 |
| 197 | 543063 | Sericealactone（5-Benzofuranacetic acid, 2,4,5,6,7,7a-hexahydro-7a-hydroxy-3,6-dimethyl-.alpha.-methylene-2-oxo-6-vinyl-, methyl ester ） | CDC25A |
| 198 | 543063 | Sericealactone（5-Benzofuranacetic acid, 2,4,5,6,7,7a-hexahydro-7a-hydroxy-3,6-dimethyl-.alpha.-methylene-2-oxo-6-vinyl-, methyl ester ） | CDC25C |
| 199 | 543063 | Sericealactone（5-Benzofuranacetic acid, 2,4,5,6,7,7a-hexahydro-7a-hydroxy-3,6-dimethyl-.alpha.-methylene-2-oxo-6-vinyl-, methyl ester ） | DCTPP1 |
| 200 | 543063 | Sericealactone（5-Benzofuranacetic acid, 2,4,5,6,7,7a-hexahydro-7a-hydroxy-3,6-dimethyl-.alpha.-methylene-2-oxo-6-vinyl-, methyl ester ） | DRD3 |
| 201 | 543063 | Sericealactone（5-Benzofuranacetic acid, 2,4,5,6,7,7a-hexahydro-7a-hydroxy-3,6-dimethyl-.alpha.-methylene-2-oxo-6-vinyl-, methyl ester ） | EDNRB |
| 202 | 543063 | Sericealactone（5-Benzofuranacetic acid, 2,4,5,6,7,7a-hexahydro-7a-hydroxy-3,6-dimethyl-.alpha.-methylene-2-oxo-6-vinyl-, methyl ester ） | FGFR1 |
| 203 | 543063 | Sericealactone（5-Benzofuranacetic acid, 2,4,5,6,7,7a-hexahydro-7a-hydroxy-3,6-dimethyl-.alpha.-methylene-2-oxo-6-vinyl-, methyl ester ） | HCRTR2 |
| 204 | 543063 | Sericealactone（5-Benzofuranacetic acid, 2,4,5,6,7,7a-hexahydro-7a-hydroxy-3,6-dimethyl-.alpha.-methylene-2-oxo-6-vinyl-, methyl ester ） | HMGCR |
| 205 | 543063 | Sericealactone（5-Benzofuranacetic acid, 2,4,5,6,7,7a-hexahydro-7a-hydroxy-3,6-dimethyl-.alpha.-methylene-2-oxo-6-vinyl-, methyl ester ） | HMOX1 |
| 206 | 543063 | Sericealactone（5-Benzofuranacetic acid, 2,4,5,6,7,7a-hexahydro-7a-hydroxy-3,6-dimethyl-.alpha.-methylene-2-oxo-6-vinyl-, methyl ester ） | HRH2 |
| 207 | 543063 | Sericealactone（5-Benzofuranacetic acid, 2,4,5,6,7,7a-hexahydro-7a-hydroxy-3,6-dimethyl-.alpha.-methylene-2-oxo-6-vinyl-, methyl ester ） | HSD17B2 |
| 208 | 543063 | Sericealactone（5-Benzofuranacetic acid, 2,4,5,6,7,7a-hexahydro-7a-hydroxy-3,6-dimethyl-.alpha.-methylene-2-oxo-6-vinyl-, methyl ester ） | HTR2A |
| 209 | 543063 | Sericealactone（5-Benzofuranacetic acid, 2,4,5,6,7,7a-hexahydro-7a-hydroxy-3,6-dimethyl-.alpha.-methylene-2-oxo-6-vinyl-, methyl ester ） | HTR7 |
| 210 | 543063 | Sericealactone（5-Benzofuranacetic acid, 2,4,5,6,7,7a-hexahydro-7a-hydroxy-3,6-dimethyl-.alpha.-methylene-2-oxo-6-vinyl-, methyl ester ） | IMPDH2 |
| 211 | 543063 | Sericealactone（5-Benzofuranacetic acid, 2,4,5,6,7,7a-hexahydro-7a-hydroxy-3,6-dimethyl-.alpha.-methylene-2-oxo-6-vinyl-, methyl ester ） | JUN |
| 212 | 543063 | Sericealactone（5-Benzofuranacetic acid, 2,4,5,6,7,7a-hexahydro-7a-hydroxy-3,6-dimethyl-.alpha.-methylene-2-oxo-6-vinyl-, methyl ester ） | MAPK1 |
| 213 | 543063 | Sericealactone（5-Benzofuranacetic acid, 2,4,5,6,7,7a-hexahydro-7a-hydroxy-3,6-dimethyl-.alpha.-methylene-2-oxo-6-vinyl-, methyl ester ） | MET |
| 214 | 543063 | Sericealactone（5-Benzofuranacetic acid, 2,4,5,6,7,7a-hexahydro-7a-hydroxy-3,6-dimethyl-.alpha.-methylene-2-oxo-6-vinyl-, methyl ester ） | NT5E |
| 215 | 543063 | Sericealactone（5-Benzofuranacetic acid, 2,4,5,6,7,7a-hexahydro-7a-hydroxy-3,6-dimethyl-.alpha.-methylene-2-oxo-6-vinyl-, methyl ester ） | PLA2G1B |
| 216 | 543063 | Sericealactone（5-Benzofuranacetic acid, 2,4,5,6,7,7a-hexahydro-7a-hydroxy-3,6-dimethyl-.alpha.-methylene-2-oxo-6-vinyl-, methyl ester ） | PPARG |
| 217 | 543063 | Sericealactone（5-Benzofuranacetic acid, 2,4,5,6,7,7a-hexahydro-7a-hydroxy-3,6-dimethyl-.alpha.-methylene-2-oxo-6-vinyl-, methyl ester ） | PTPN1 |
| 218 | 543063 | Sericealactone（5-Benzofuranacetic acid, 2,4,5,6,7,7a-hexahydro-7a-hydroxy-3,6-dimethyl-.alpha.-methylene-2-oxo-6-vinyl-, methyl ester ） | RPS6KA5 |
| 219 | 543063 | Sericealactone（5-Benzofuranacetic acid, 2,4,5,6,7,7a-hexahydro-7a-hydroxy-3,6-dimethyl-.alpha.-methylene-2-oxo-6-vinyl-, methyl ester ） | SIGMAR1 |
| 220 | 543063 | Sericealactone（5-Benzofuranacetic acid, 2,4,5,6,7,7a-hexahydro-7a-hydroxy-3,6-dimethyl-.alpha.-methylene-2-oxo-6-vinyl-, methyl ester ） | SLC6A4 |
| 221 | 543063 | Sericealactone（5-Benzofuranacetic acid, 2,4,5,6,7,7a-hexahydro-7a-hydroxy-3,6-dimethyl-.alpha.-methylene-2-oxo-6-vinyl-, methyl ester ） | STK3 |
| 222 | 543063 | Sericealactone（5-Benzofuranacetic acid, 2,4,5,6,7,7a-hexahydro-7a-hydroxy-3,6-dimethyl-.alpha.-methylene-2-oxo-6-vinyl-, methyl ester ） | TTK |
| 223 | 564243 | 8-Methylenebicyclo[4.2.0]oct-4-en-3-one | CYP17A1 |
| 224 | 564243 | 8-Methylenebicyclo[4.2.0]oct-4-en-3-one | FABP1 |
| 225 | 249955570 | 7,8-Diazabicyclo[4.2.2]deca-2,4,7,9-tetraen-7-oxide | TRPA1 |
| 226 | 561486 | Tricyclo[4.2.1.0(2,5)]non-7-en-3-one | ADH1A |
| 227 | 561486 | Tricyclo[4.2.1.0(2,5)]non-7-en-3-one | ADH1B |
| 228 | 561486 | Tricyclo[4.2.1.0(2,5)]non-7-en-3-one | ADH1C |
